# Making atomistic materials calculations accessible with the AiiDAlab Quantum ESPRESSO app

**DOI:** 10.1038/s41524-025-01936-4

**Published:** 2026-02-03

**Authors:** Xing Wang, Edan Bainglass, Miki Bonacci, Andres Ortega-Guerrero, Lorenzo Bastonero, Marnik Bercx, Pietro Bonfà, Roberto De Renzi, Dou Du, Peter N. O. Gillespie, Michael A. Hernández-Bertrán, Daniel Hollas, Sebastiaan P. Huber, Elisa Molinari, Ifeanyi J. Onuorah, Nataliya Paulish, Deborah Prezzi, Junfeng Qiao, Timo Reents, Christopher J. Sewell, Iurii Timrov, Aliaksandr V. Yakutovich, Jusong Yu, Nicola Marzari, Carlo A. Pignedoli, Giovanni Pizzi

**Affiliations:** 1 PSI Center for Scientific Computing, Theory and Data, Villigen PSI, Switzerland; 2 National Centre for Computational Design and Discovery of Novel Materials (MARVEL), Villigen PSI, Switzerland; 3 nanotech@surfaces Laboratory, Empa-Swiss Federal Laboratories for Materials Science and Technology, Dübendorf, Switzerland; 4 U Bremen Excellence Chair, Bremen Centre for Computational Materials Science, and MAPEX Center for Materials and Processes, University of Bremen, Bremen, Germany; 5 Dipartimento di Scienze Fisiche, Informatiche, Matematiche (FIM), Università di Modena e Reggio Emilia, Modena, Italy; 6 Nanoscience Institute, National Research Council (CNR-NANO), Modena, Italy; 7 Department of Physics and Earth Sciences, University of Parma, Parma, Italy; 8 Theory and Simulation of Materials (THEOS), École Polytechnique Fédérale de Lausanne, Lausanne, Switzerland; 9 Center for Computational Chemistry, School of Chemistry, University of Bristol, Bristol, UK

**Keywords:** Materials science, Physics

## Abstract

Despite the wide availability of density functional theory (DFT) codes, their adoption by the broader materials science community remains limited due to challenges such as software installation, input preparation, high-performance computing setup, and output analysis. To overcome these barriers, we introduce the Quantum ESPRESSO app, an intuitive, web-based platform built on AiiDAlab that integrates user-friendly graphical interfaces with automated DFT workflows. The app employs a modular Input-Process-Output model and a plugin-based architecture, providing predefined computational protocols, automated error handling, and interactive results visualization. We demonstrate the app’s capabilities through plugins for electronic band structures, projected density of states, phonon, infrared/Raman, X-ray and muon spectroscopies, Hubbard parameters (DFT+*U*+*V*), Wannier functions, and post-processing tools. By extending the FAIR principles to simulations, workflows, and analyses, the app enhances the accessibility and reproducibility of advanced DFT calculations and provides a general template to interface with other first-principles calculation codes.

## Introduction

Density-functional theory (DFT) simulations play a pivotal role in condensed matter physics and chemistry by providing insights and predictions for electronic structure and properties of materials at the atomistic level^1,2^. These simulations enable researchers to predict a wide range of properties—such as electronic, magnetic, optical, mechanical, and thermal behavior—aiding in the design of novel materials for applications across diverse fields, including semiconductors, catalysts, energy storage systems, quantum materials, nanotechnology, and more^3^.

A multitude of powerful and widely used simulation codes have been developed in the past decades^4^ and cross-verified^5,6^, to perform these calculations precisely and efficiently. Despite these advances, performing DFT calculations remains a complex, multi-stage process that demands significant time and effort, particularly for non-specialists. By non-specialists, we refer to researchers whose primary expertise lies outside computational materials science, such as experimentalists, industrial R&D engineers, educators, and students. These users often face steep learning curves due to obstacles including: (1) software installation and configuration, (2) code-specific input file preparation, (3) parameter tuning, (4) results interpretation, (5) setup and utilization of high-performance computing (HPC) resources, and (6) validation and benchmarking against experimental data or established theoretical values. Furthermore, many property calculations require orchestrating long sequences of interdependent simulations—sometimes numbering in the tens or even hundreds. For instance, simulating phonon properties requires performing total energy calculations for each unique finite displacement (in supercells) as well as, if long-range dipole corrections are needed, for each applied finite electric field (in the unit cell). Even seasoned computational materials scientists (whom we will refer to as specialists in the following) routinely encounter such time-consuming, error-prone workflows that distract them from scientific insight.

Consequently, there is a growing demand for platforms that simultaneously (1) lower the entry barrier for non-specialists, enabling them to perform reliable simulations with minimal friction (for example, making advanced workflows accessible to experimentalists to help them interpret experimental results or guide the design of new experiments); and (2) provide specialists with robust, reproducible, and extensible infrastructures and advanced interactive analysis to enhance productivity in their simulations, as well as to allow them to incorporate their domain expertise into the platform, making it accessible to non-specialists. Achieving this goal requires a combination of: (1) ensuring that simulation codes use optimal computational settings with minimal user intervention; (2) implementing workflows that can detect and recover from common failures (e.g., switching to more robust algorithms in cases of convergence issues); (3) providing user-friendly user interfaces (UIs) to assist non-specialists in preparing inputs, submitting and monitoring workflows, and analyzing results; and (4) enabling easy sharing and reusing of data to foster transparency and collaboration.

Over the past decade, formal workflows have become increasingly important in computational materials science, enabling the automation of simulations and supporting large-scale, high-throughput studies that generate extensive databases of material properties^7–15^. Available workflow frameworks include AFLOW^16^, AiiDA^17–19^, ASR+ASE+myqueue^20,21^, Atomate+FireWorks^22,23^, Covalent^24^, Jobflow^25^, HTTK^26^, MISPR^27^, Pyiron^28^, and QMPY^29^, among others. These frameworks facilitate researchers in managing automated sequences of simulations and often promote the FAIR (Findable, Accessible, Interoperable, and Reusable) data principles^30^, making it easier to share and reuse data generated by workflows among researchers, thus advancing the collective understanding of material behavior. One noteworthy advancement in this field has been the development of common workflows for computing material properties across different quantum engines^20,31–33^. These workflows provide a standardized approach to property calculations, improving reproducibility and facilitating direct comparison and verification between different DFT implementations^6^. Despite these advancements, existing platforms often require users to formulate workflows through scripting, necessitating programming skills that many non-specialists may not master. Additionally, these frameworks do not address the practical challenges, such as software installation and computational resource setup. Therefore, despite the advantages of workflows, significant barriers remain for non-specialists wishing to leverage these tools.

Graphical user interfaces (GUIs) could remedy this situation. However, developing GUIs for workflows traditionally requires in-depth knowledge of both workflow engines and graphical programming languages. Mature commercial offerings, such as AMS^34^, ASAP^35^, Mat3ra^36^, Materials Studio^37^, Materys^38^, MedeA^39^, QuantumATK^40^, Schrödinger^41^, and SimStack^42^ have successfully tackled many of the practical challenges we outline above. Nevertheless, these platforms are often proprietary, and their closed-source nature could limit the ability of users to inspect, modify, and integrate the underlying code into the open-source ecosystem, thereby constraining transparency, reproducibility, and full compliance with FAIR principles. On the open-source front, Quantum ESPRESSO (QE) provides its own GUI, PWgui^43^, and several third-party tools have also been developed, such as BURAI^44^, which are excellent for generating input files for single calculations. Other codes, like GPAW^45^, also provide dedicated GUIs. While invaluable, these tools are typically limited to specific codes and often focus on setting up individual calculations rather than managing complex, multi-stage workflows. Other frameworks—such as CINEMAS^46^ and ALKEMIE^47^—have emerged, providing user-friendly GUIs and integrated environments for advanced materials simulations. However, these solutions generally require a local installation, highlighting the ongoing need for more flexible, web-based platforms that can accommodate both local and cloud-based usage while remaining widely accessible to the research community.

In our previous work, we developed AiiDAlab^48^, a web-based platform that enables computational scientists to package scientific workflows and computational environments into user-friendly applications (apps). AiiDAlab integrates AiiDA workflows with graphical interfaces (based on Jupyter notebooks) for execution and data analysis, making them accessible to a broader audience. In this work, leveraging the AiiDAlab platform, we identify a common structure when designing an app and GUI for DFT calculations, and develop a generic system that can serve as a template for other simulation codes. We demonstrate this concept by implementing a feature-rich app specifically designed for QE^49–51^, an open-source and widely used DFT simulation package^4^. In the following sections, we describe the architecture and general structure of the app, presenting some implementation details including the integration with AiiDA and AiiDAlab. We then demonstrate how it addresses the challenges faced by non-specialist users in performing DFT simulations by showcasing the app’s features through example use cases. These features leverage several AiiDA workflows recently developed for computing relevant materials properties (ranging from electronic structure calculations to vibrational and spectroscopic properties) and for supporting and automating advanced computational methods (such as DFT+*U*+*V* and Wannier functions).

## Results

### Architecture overview

**Fig. 1:**
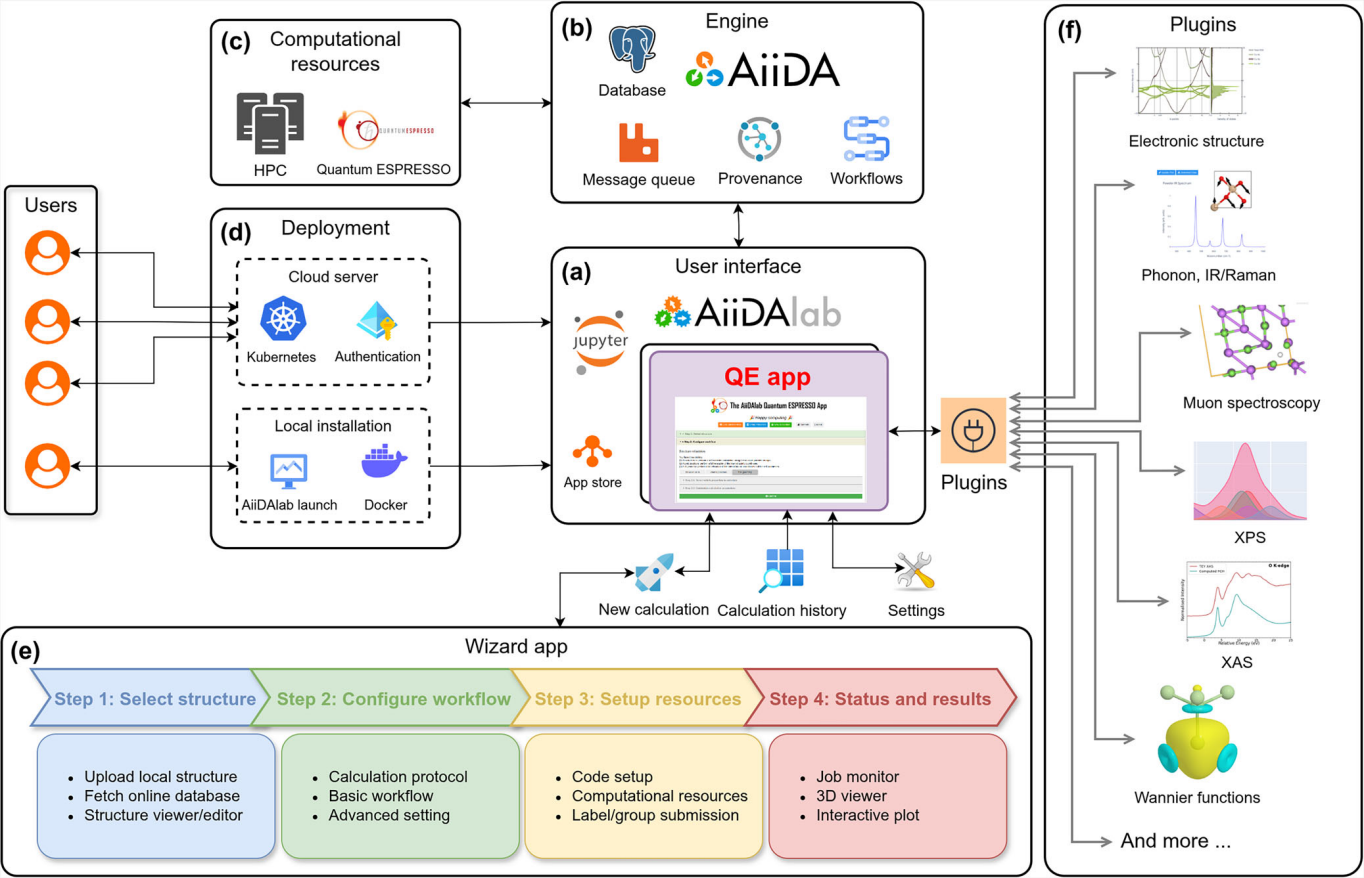
Overview of the QE app’s architecture. **a** The UI is built within the AiiDAlab environment using Jupyter notebooks and the appmode plugin, providing an intuitive front end for simulation setup and results analysis. **b** The backend engine is powered by AiiDA, which handles workflow orchestration, data management, and provenance tracking. **c** The app connects to external high-performance computing (HPC) resources for running simulations. **d** The app can be deployed on cloud-based deployments using Kubernetes and authentication layers, or locally using the AiiDAlab launch tool or Docker. **e** A modular wizard UI guides users through the four main steps of the simulation process. **f** The plugin system enables extensibility by allowing users to activate specific property calculations or post-processing tools, such as electronic structure, vibrational properties (IR/Raman), muon spectroscopy and resting sites, XPS, XAS, Wannier functions and more. The full list of available plugins is discussed later in the text.

The QE app architecture aims to provide a turn-key solution for materials scientists to run DFT calculations seamlessly, addressing key challenges outlined in the introduction. As illustrated in Fig. 1, the app consists of several key components to handle different aspects of the simulation workflow: (a) a user interface (UI) built within the AiiDAlab ecosystem using Jupyter notebooks and the appmode plugin^52^, (b) an AiiDA-based backend engine responsible for workflow management and data provenance that interacts with (c) external HPC resources, (d) flexible deployment options, including both cloud-based and local installations, (e) a “wizard” app guiding users through the simulation pipeline, and (f) a plugin system for extensibility, providing access to several advanced workflows to compute different material properties, combined with specialized post-processing tools.

One of the key challenges in designing the QE app is that different material properties require distinct inputs, workflows, and outputs. A straightforward but suboptimal approach would be to create a dedicated UI or sub-app for each property, but this leads to a fragmented user experience and increased maintenance complexity. In contrast, this app adopts a unified design strategy that enables users to perform calculations of different material properties using QE within a single, coherent interface. At the core of the app’s UI design is the Input-Process-Output (IPO) model^53^, which is widely used in workflow management systems, where it provides a clear and organized approach to handling tasks, ensuring systematic processing across different stages^54,55^. The app implements the IPO model at two interconnected levels. On the backend, a top-level AiiDA WorkChain orchestrates property-specific sub-workflows, managing inputs, processes, and outputs in a standardized way. A detailed schematic of this IPO workflow architecture is provided in the **Input-Process-Output model** section of the Supplementary Information. On the frontend, this logic is mirrored by a wizard UI that guides users through the simulation process in a structured and intuitive way (see Fig. 1e). The wizard ensures that all necessary inputs are provided before proceeding to the next step, but also allows users to revisit and modify previous steps if needed, offering both flexibility and control during the setup process. The details of the wizard UI are provided in a later section.

In academic development projects, rapid prototyping often requires incorporating new algorithms and protocols into existing workflows. The QE app addresses this need with a plugin-based architecture that allows flexible addition of new functionalities. Material property calculations, such as band structures or projected density of states (PDOS), are implemented as plugins, each operating as a self-contained unit within the IPO model, managing its own input, process, and output. The main app provides a unified API and mounts the components of the plugins, ensuring seamless integration. This design preserves a consistent interface across all functionalities, allowing the main application to focus on core features shared by all plugins. It also simplifies the work of plugin developers, who can build and test their components independently, without needing to modify the core application. Plugins can be developed and maintained either within the main code base or as standalone packages. By extending functionality through plugins without altering the core structure, the QE app maintains a single, streamlined interface for setup, execution, and analysis, reducing the learning curve and delivering a smooth, consistent user experience.

### App accessibility

The QE app can be used both locally and via the cloud, offering flexibility for different user needs and environments. For local workstations, the app can be installed via the aiidalab-launch utility (see the **Code availability** section), which automates the process of retrieving the appropriate Docker image and launching a containerized instance of AiiDAlab with the QE app pre-configured. This option enables users to run the application in a fully isolated and reproducible environment on their own machines.

For cloud access, AiiDAlab’s centralized Software-as-a-Service (SaaS) model allows users to access the platform via a web browser without the need for local software installations. This model supports scalable deployment, from individual users and small research groups to larger institutional infrastructures. We provide a publicly accessible demonstration instance of the QE app at (https://demo.aiidalab.io). The server is deployed via a dedicated Kubernetes infrastructure on the Microsoft Azure service and comes with the app and its core plugins pre-installed. Users can readily explore the app’s functionality, inspect pre-computed examples, and submit test calculations. This public demo is currently maintained under the NCCR MARVEL. The goal of this demo is to facilitate first-time users in getting a first impression of the functionality of the app and of the platform without the need of any local installation. Should the public server become unavailable in the future, the app’s continued accessibility is ensured through the local installation option via aiidalab-launch described earlier, which provides a permanent way for users to run the tool on their own machines.

### Leveraging AiiDA for workflow management

Workflows help standardize simulations, minimize manual intervention, and improve reproducibility, making them an essential component of the app’s turn-key solutions. To manage its calculation workflows, the QE app leverages the AiiDA workflow management system, which serves as the backend engine for job execution, data provenance, and automated workflow coordination. AiiDA manages communications with HPC machines, transferring files, interacting with the job scheduler, and submitting and monitoring calculations. Besides, AiiDA supports a broader ecosystem of community-maintained plugins, available through an official registry (https://aiidateam.github.io/aiida-registry/), which covers a wide range of simulation codes, including both calculation interfaces and automated workflows.

The app uses the aiida-quantumespresso plugin package (see the **Code availability** section), which provides a collection of pre-configured AiiDA calculation plugins to prepare the input and parse the output of several of the QE executables. These calculation classes serve as building blocks for more complex workflows that link multiple computations together, enabling users to perform sophisticated simulation tasks with minimal manual input.

AiiDA also supports automatic restarts and error handling mechanisms. Building on this, the aiida-quantumespresso plugin implements specific recovery strategies tailored to QE calculations. For example, if a calculation fails to reach electronic convergence, the workflow can automatically adjust key parameters, such as reducing the mixing ratio for self-consistent iterations. Similarly, if a job is interrupted due to exceeding the allocated walltime, it can be resumed cleanly using the last output structure and charge density. These recovery strategies are embedded in the workflow logic and require no user intervention, reducing manual troubleshooting and increasing the reliability of complex simulations. In addition, AiiDA implements a caching mechanism that allows the reuse of results from previous calculations submitted with identical inputs, thus avoiding redundant computations and promoting consistency across multiple runs. To streamline the process of preparing simulations, the app exposes other utilities from aiida-quantumespresso to facilitate simulation setup and customization, for example, to automate **k**-point mesh generation tailored to the size of the selected system.

### QE app wizard UI

The app’s wizard UI serves as a guide for users as they construct and submit a workflow. Throughout the process, the wizard ensures that all necessary information is provided before progressing. Each step in the wizard consists of multiple panels that allow users to refine their input as needed. Prior to submission, users can also return to a previously confirmed step to modify any input data. The UI divides the entire process into four main steps.

**Fig. 2:**
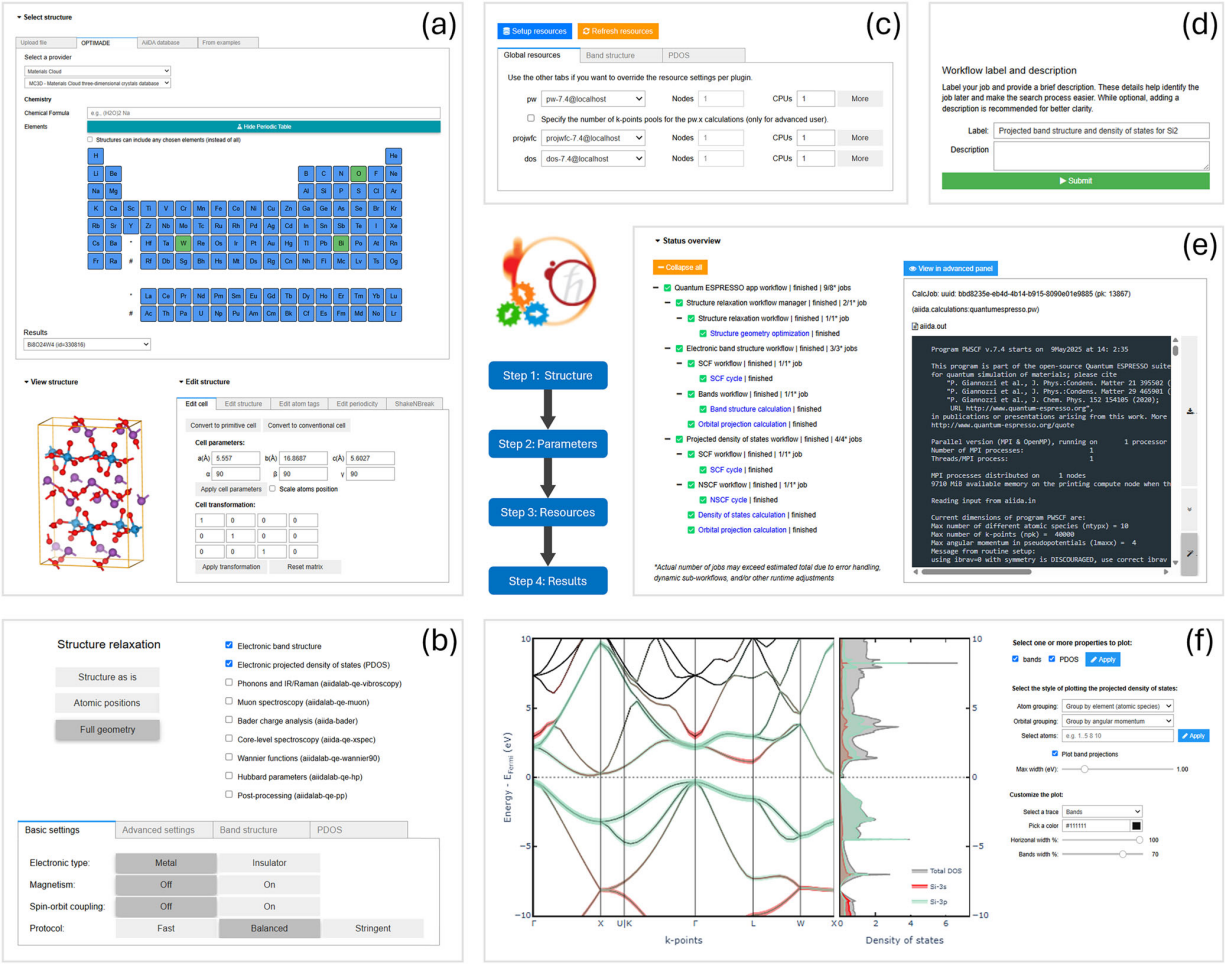
The QE app’s wizard UI steps. **a** structure selection step, where users can select, view, and edit the input structure of the calculation; **b** workflow configuration step, where users specify the main workflow steps (structure relaxation and properties to compute) and customize the parameters of each of them; **c**, **d** resource selection and workflow submission step, where users select codes and computational resources, provide optional metadata, and submit the workflow; and **e**,**f** monitoring and analysis step, where users can monitor the running calculations, plot results from completed calculations, and customize plots interactively.

The first step is selecting a structure. This step includes tools to select, view, and modify the input structure (Fig. 2a). Users can upload their own structure (in any standard format, such as XYZ, CIF, etc.), or choose a structure from online databases compliant with the OPTIMADE standard^56,57^ (such as the Materials Project^8^, the Materials Cloud^58^ and the Alexandria Materials Database^59,60^), from their own local AiiDA database, or from a set of example structures. The structure viewer allows for inspection, providing an overview of structure properties (cell parameters, volume, space/point group) and with a set of tools to interact with and/or manipulate the atomistic model: edit the structure cell (primitive, conventional, and supercell transformations), periodicity, (surfaces, molecule definition), and atoms (removal, tagging). Other editing features include structural modifications, such as introducing point defects in 2D materials and applying bond distortions or random displacements to explore metastable configurations of point defects, following the approach implemented in the ShakeNBreak package^61^.

The second step is configuring the workflow. With an input structure selected, users can now configure the workflow, optionally including a structure geometry optimization step, selecting from a host of properties of interest to compute (see **Plugins** section, also in Supplementary Information), and customizing calculation parameters (Fig. 2b). To simplify this process, the app splits the parameter customization step into basic, advanced, and property-specific panels. The basic panel streamlines the process by abstracting much of the complexity via a set of top-level controls, including a protocol selector to set most input parameters balancing calculation speed and accuracy^62^. Full flexibility in customizing calculation inputs is retained via the advanced panel. In this panel, the user can choose among various pseudopotential families, including the Standard Solid-State Pseudopotentials (SSSP; efficiency or precision)^63^ and PseudoDojo (standard or stringent)^64^, or alternatively upload custom pseudopotential files directly via the GUI. Further details of each panel are provided in the Workflow parameter settings panels section of the Supplementary Information.

The third step is choosing computational resources and submitting the workflow. Users proceed to choose the computational resources to use by AiiDA to run the calculation (Fig. 2c). The app provides a straightforward UI for selecting AiiDA Code instances—references to executables on local or remote machines—and specifying the computational resources to be allocated per code (e.g., nodes, CPUs). Additional codes may be set up in a separate notebook (see the Computational resources setup section of the Supplementary Information). Once the resources have been chosen, users may customize the auto-generated workflow label and optionally provide a description to attach to the workflow (Fig. 2d). When ready, users can submit the workflow. AiiDA will handle submission in the background.

The fourth step is monitoring and viewing workflow results. After submitting a workflow, the app redirects users to the status panel of the results step (Fig. 2e), where they can monitor the calculations in real-time. Users can switch to the summary panel to inspect a summary of the selected inputs. As calculations complete, results from each calculation submitted by the app’s workflow are made available for analysis in dedicated sub-panels of the results panel (Fig. 2f). Lastly, when the workflow is finished, users can download from the summary panel either a zip file including the raw input and output data of all workflow steps, or directly a single AiiDA archive (including the full provenance graph) for further inspection or sharing. The latter option (AiiDA archive) allows users to import the workflow into another AiiDAlab instance and continue inspecting the results via the QE app GUI.

The app’s plugin-based architecture provides several entry points to extend the core UI described above. Plugin developers can introduce additional UI components, including structure importers and editors, property-specific parameter settings panels, computational resource panels to override global resources per property-specific code, and property-specific results panels. The following section provides examples of such plugin components.

### Plugins: overview

The QE app’s plugin-based architecture provides a flexible framework for extending the platform with specialized functionalities, ranging from electronic-structure analysis to advanced spectroscopic techniques. Each plugin incorporates its own workflows and UI components, yet remains fully integrated within the app’s unified IPO model and the broader AiiDAlab environment. This modular design not only streamlines maintenance and testing but also encourages community-driven extensions. Below, we highlight the key aspects of two plugins, one provided as a core plugin included in the app’s code base (electronic structure) and one developed as an external plugin (vibrational spectroscopies). We also provide a short summary for each of the currently supported app plugins. The plugin packages are listed in the **Code availability** section.

### Plugins for the electronic structure: band structures and (projected) densities of states

Electronic band structure theory is fundamental for interpreting material's behavior, including electrical transport and optical properties, making it a cornerstone of solid-state physics^65^. In the QE app, band-structure calculations are streamlined through a dedicated plugin that guides the users from processing to final visualization. The process begins with supplying an initial crystal structure, which can be either a representation of an experimentally known structure or a computationally optimized one. The app automatically defines the band-structure path based on the structure. For 3D materials, the app leverages existing tools—such as seekpath^66^—to standardize the lattice and identify a **k**-point path based on well-known symmetry lines^67^. In low-dimensional cases (e.g., 2D monolayers), the app detects the system symmetry and selects the appropriate reciprocal paths that capture the most relevant dispersion features.

**Fig. 3:**
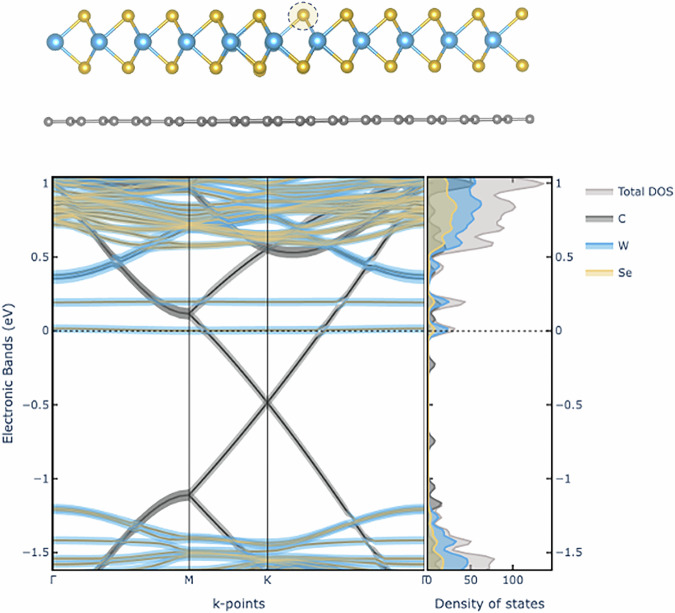
Schematic illustration of a selenium (Se) vacancy in a WSe_2_/graphene heterojunction (top) and the corresponding projected band structure and projected density of states (PDOS) (bottom), computed using the QE app with spin-polarized DFT using the PBE exchange-correlation functional^116^, and including SOC. This study explores the hybridization of Se vacancy states with graphene and their interactions. The in-gap defect states, which are non-dispersive and exhibit a gap due to spin-orbit coupling (SOC) effects, are located between the conduction and valence bands of WSe_2_, as discussed in ref. ^117^.

Beyond computing the band energies, the plugin incorporates projected band structure analysis, also known as “fat bands”, which quantifies the contribution of specific atomic orbitals to each energy level; an example is shown in Fig. 3f. These projections help reveal crucial effects, such as orbital hybridization, localized defect states, or spin-splitting phenomena^68^. In addition to fat bands, where projection onto atomic orbitals is studied only along the high-symmetry paths in reciprocal space where the band structure is computed, PDOS calculations provide an additional perspective on the electronic structure of materials by revealing how specific atoms or orbitals contribute to the total density of states (DOS), e.g. helping in identifying which atomic orbitals dominate states near the Fermi level, thereby offering insights into the origins of electronic, magnetic, or optical properties. In the QE app, the PDOS workflow integrates four core steps: self-consistent field (SCF), non-self-consistent field (NSCF), total DOS, and PDOS, into a streamlined sequence that automatically generates necessary wavefunction files and appropriately handles large intermediate data. In particular, orbital-projected DOS calculations decompose the total DOS into site- and orbital-specific contributions, highlighting the role of, for instance, *d*-orbitals in transition-metal oxides or *p*-orbitals in organic semiconductors. Moreover, both the band structure and PDOS plugins support spin–orbit coupling (SOC) calculations, enabling an accurate description of relativistic effects in materials where SOC plays a significant role, such as heavy-element semiconductors and topological insulators, and allowing users to analyze SOC-induced modifications in the DOS and PDOS. Within the standard app UI, users can also configure advanced parameters, such as **k**-point densities, smearing schemes, or energy ranges. This flexibility is particularly important for materials where fine spectral features can strongly influence theoretical predictions of conductivity or band alignments. The final PDOS data are presented via interactive plots, allowing for immediate visual inspection of peaks and energy offsets relative to the Fermi level. The PDOS can also be displayed in tandem with the band structure, further clarifying which states govern conduction or valence behavior, as we illustrate in Fig. 3. The interactive panel of the plugin enables users to adjust visual elements, isolate specific states, and export publication-quality figures or the underlying numerical data. By automating complex workflows in a reproducible environment, this plugin significantly reduces the computational barrier for advanced electronic structure studies of complex materials.

### Plugins for vibrational spectroscopies: phonons and Raman/Infrared spectra

Vibrational spectroscopies are among the most powerful methods for materials structure investigation, via fingerprint atomic vibrations probed by using several types of radiation, such as light. The simulation of vibrational properties in the QE app is available through the aiidalab-qe-vibroscopy plugin. The app has been successfully applied to investigate the vibrational and optoelectronic properties of BaZrS_3_, highlighting its practical utility in ongoing research and materials characterization efforts^69^. This is accomplished via the specific workflow, called VibroWorkChain, which orchestrates the submission of AiiDA workflows provided by the aiida-vibroscopy^70^ and aiida-phonopy^70–73^ plugins. Specifically, phonon properties are obtained using a finite-difference approach^67^ through multiple SCF calculations, involving finite atomic displacements from equilibrium positions in supercells. Large enough supercells are needed to accurately interpolate phonon dispersions, equivalent to using dense **q**-point meshes in density-functional perturbation theory (DFPT)^74^. If long-range non-analytic corrections (NACs) are required, the workflow will also compute dielectric properties via finite applied electric fields^75^ simulations. This approach differs from the one implemented in the Phonon code of the QE package^74^, and enables calculations with any functional, such as hybrid functionals or DFT+*U*(+*V*)^70^. Various types of simulations can be performed based on the desired outcome: (1) full characterization of phonon properties, including phonon bands, phonon PDOS, thermodynamics, and infrared (IR) and Raman spectra; (2) phonon properties for non-polar materials (i.e., NACs are not computed); (3) IR/Raman spectra from *Γ*-point normal modes of the unit cell; (4) dielectric properties (dielectric and Raman tensors, Born effective charges). As shown in Supplementary Fig. S4, options are provided to users by means of a drop-down menu, together with options to select (or automatically determine) the supercell size needed for finite atomic displacements and the symmetry tolerance used to detect unique atomic displacements (thus reducing the cost of the simulation). As a rule of thumb, we suggest a supercell with lattice parameters of at least 15 Å, which is typically enough to capture all the relevant atomic displacements to be considered in the phonon simulation, i.e., for force-constant matrix elements to decay to zero for atom pairs beyond this distance. An estimate of the number of finite displacement supercells can be performed, providing an indication of the number of simulations that will be submitted, and thus of the total computational cost.

If phonons are computed (i.e., if users select one of the first two calculation options), the inelastic neutron scattering (INS) dynamical structure factor can additionally be computed as a live post-processing functionality in the output panel. Specifically, INS observables for both single-crystal and powder samples are calculated by means of the Euphonic package^76^ in a format directly comparable with experimental spectra. Users also have the possibility to provide a specific reciprocal space path or plane to facilitate comparison of the simulation results with a specific experimental setup and measurements.

**Fig. 4:**
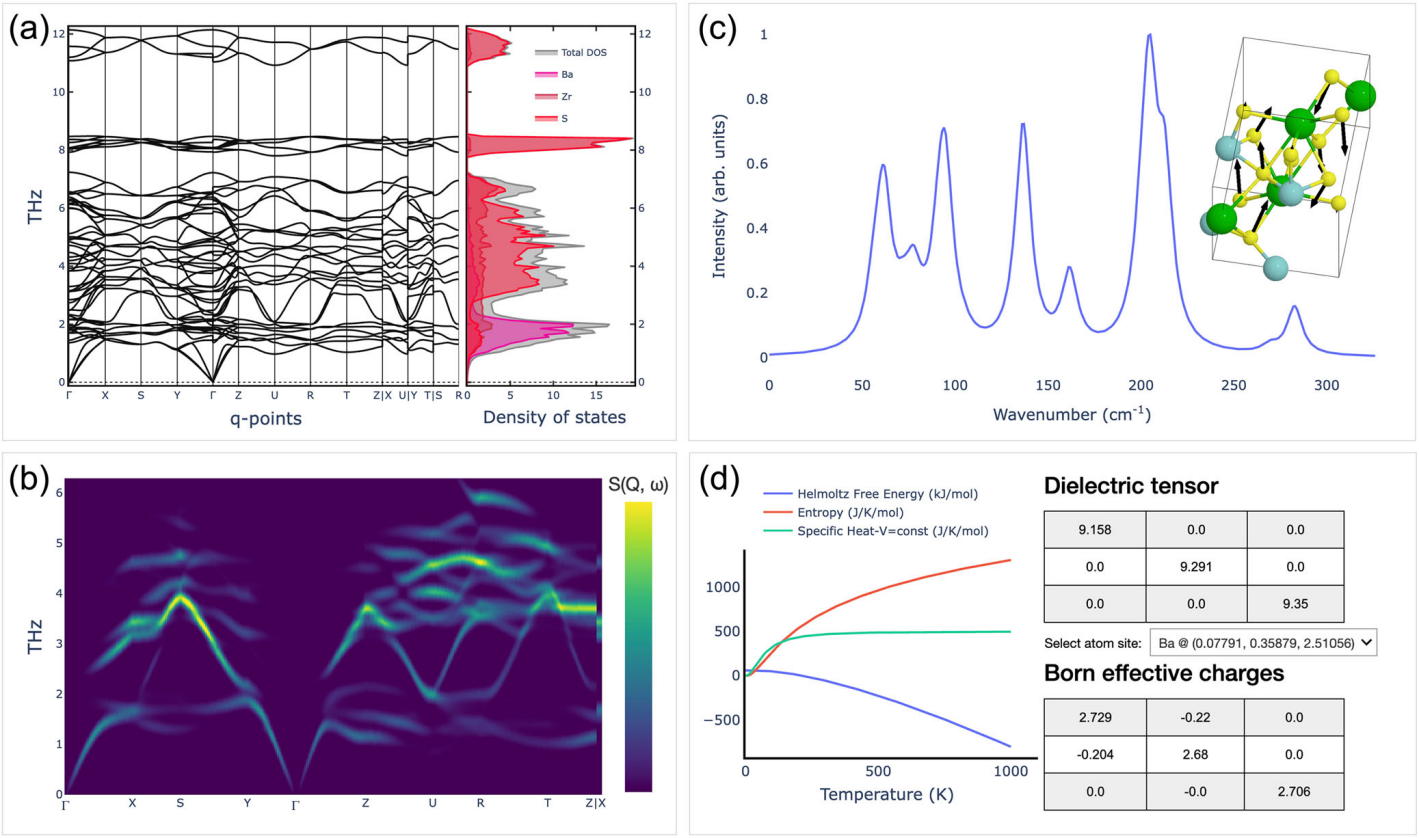
Results panels for the aiidalab-qe-vibroscopy plugin. **a** Phonon dispersion plot for BaZrS_3_, together with the phonon DOS (aligned in energy with respect to the phonon bands). **b** Computed INS spectrum. **c** Single crystal Raman spectrum and modes animation. **d** Thermodynamic properties, dielectric tensor and Born effective charges.

Selected screenshots of the plugin interface are shown in Fig. 4 for the previously mentioned BaZrS_3_ system^69^, showing that the results tab is subdivided into different sub-tabs, each of them dedicated to a specific output of the simulations: (i) phonon band structure, phonon PDOS and thermodynamic quantities, see panel (a); (ii) INS results, to be computed live, see panel (b); (iii) IR and Raman active modes, together with their interactive 3D animations, see panel (c); (iv) thermodynamic and dielectric properties, see panel (d). All the data shown in the panels can be downloaded and analyzed with additional external tools, if needed.

### Additional plugins

**Fig. 5:**
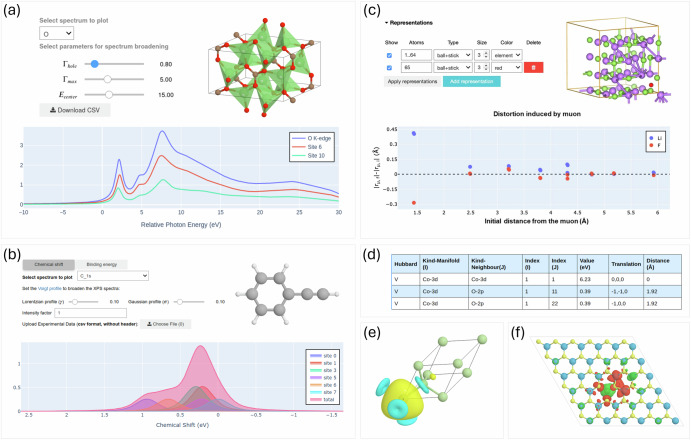
Overview of result panels from various plugins. **a** XAS plugin showing the O K-edge XANES spectrum of crystalline Li_2_CO_3_, including site-resolved and total contributions. **b** XPS plugin applied to phenylacetylene (C_8_H_6_) in gas phase, illustrating the C 1s core level shifts and their relation to atomic environments. **c** Muon spectroscopy plugin showing a candidate muon site in LiF and the distortions induced by the muon to the hosting lattice. **d** Hubbard *U* + *V* plugin calculating on-site and inter-site interactions in LiCoO_2_, including a table of computed parameters and their atomic associations. **e** The Wannier function plugin showing isosurfaces of one of the maximally localized Wannier functions in gallium arsenide. f Post-processing plugin visualizing spin density data in a sulfur vacancy in a monolayer of MoS_2_.

Beyond the two headline examples, the app supports a growing suite of specialized plugins designed to automate and visualize advanced materials simulations. Below, we summarize the key functionalities and materials properties addressed by each plugin. Representative result panels from these plugins are shown in Fig. 5, while setup options and further technical details are provided in the **Plugins** section of the Supplementary Information.

The XAS plugin facilitates the computation of X-ray absorption near-edge structure (XANES), a technique sensitive to the local chemical environment of absorbing atoms^77^. The plugin is based on a reciprocal-space pseudopotential scheme, as implemented in the xspectra.x code^78,79^ of QE, and accounts for different core-hole treatments, symmetry-analysis for inequivalent site selection, and lifetime broadening^79^. Users can select the absorbing element and inspect both total and site-resolved XANES spectra. Interactive controls allow adjusting broadening parameters and exporting spectra for further analysis.

The XPS plugin enables the calculation of core-level binding energies using a total-energy difference approach^80–82^, widely used to interpret experimental XPS spectra and identify chemical shifts across atomic sites. Users can select target atoms, specify core-hole pseudopotentials, and set spectral broadening parameters. The results panel displays both the computed binding energies and their site-resolved chemical shifts in an interactive table and spectrum viewer, allowing comparison with experimental reference data.

The muon spectroscopy plugin targets the prediction of muon stopping sites and their interaction with the magnetic environment, crucial for interpreting muon spin relaxation (*μ*SR) experiments^83^. By simulating a dilute positive muon in a material using DFT+*μ*^84–86^, users can identify likely stopping sites and compute associated local fields. The setup panel lets users configure the supercell size, charge state, and number of trial sites (inputs for the aiida-muon plugin^87,88^). The results include a ranked list of candidate sites with respect to relative total energy, 3D site visualization and, optionally, the muon spin polarization functions computed for different crystal orientations via the UNDI package^89^.

The Wannierization plugin automates the generation of maximally localized Wannier functions (MLWFs), used to analyze bonding characters, build tight-binding models, and study topological invariants^90–92^. Users can choose between automated Wannierization schemes [e.g., the selected columns of the density matrix (SCDM) method^93,94^ or the projectability disentanglement Wannier functions (PDWF) method^95^] and adjust the energy windows^95^. It is also possible to compute the Fermi surface using Wannier interpolation, allowing for dense Brillouin zone sampling (k-point distance down to a few hundredths of reciprocal angstrom). For the generated Fermi surface, it is possible to calculate de Haas–van Alphen (dHvA) oscillation frequencies, facilitating direct comparison with experimental measurements^96,97^. The result panel compares DFT and Wannier-interpolated bands, reports total and component spreads, offers a 3D visualization of individual Wannier functions with atoms, and provides a plot with computed dHvA frequencies. Files containing the tight-binding model in the MLWF basis set and the Fermi surface are available in the “Downloads” section of the result panel.

Accurate determination of on-site *U* and inter-site *V* Hubbard corrections is essential for treating transition-metal and rare-earth compounds containing partially filled and localized *d* and/or *f* electrons^98–101^. The Hubbard parameters plugin interfaces with the aiida-hubbard workflows^102^, which efficiently orchestrate DFPT-based calculations^103,104^ to compute these parameters from first principles using the hp.x code of QE^105^. Users can toggle between “one-shot” or “self-consistent” modes, choose atom pairs for inter-site corrections, and specify convergence thresholds. Results include a summary table of the computed Hubbard parameters and an interactive structure viewer linking each value to its corresponding site or atom pair.

The post-processing plugin enables real-space visualization of properties, such as charge and spin densities, wavefunctions, electrostatic potential, integrated local density of states (ILDOS), local density of states at specific energies, and STM images using the pp.x and Critic2 codes^106^. Users can select previously completed calculations, choose the target quantity, and define isosurface or plane-plot parameters. Results are displayed using an integrated 3D viewer powered by weas-widget^107^, providing an intuitive way to correlate electronic structure features with spatial distributions.

The Bader charge analysis plugin, by partitioning the charge density into atomic basins (based on the Bader code^108^), provides chemically meaningful net atomic charges—useful for understanding charge transfer, oxidation states, or catalysis. The plugin processes charge density data from completed calculations and presents the atomic charges in a sortable, interactive table alongside the structural model.

### App utilities

Apart from the core and plugin functionalities encapsulated in the wizard UI, the QE app also provides a set of utility pages for the following operations. This includes browsing calculation history, where users can browse, filter, and manage all previously submitted jobs (see Supplementary Fig. S15); installing app plugins, which allow users to find, install, and manage plugins, customizing their computational environment as needed (see Supplementary Fig. S16); configuring computational resources, where users can define local and remote machines and codes, as well as browse (and optionally disable) existing codes (see Supplementary Fig. S17); downloading example calculations, enabling users to import example AiiDA archives into their database with one click. We provide a host of examples spanning core and plugin functionality, covering most common use cases (see Supplementary Fig. S18). Further details on each utility are provided in the **Utilities** section of the Supplementary Information.

## Discussion

The QE app and its underlying AiiDA ecosystem demonstrate in practice how the FAIR principles can be extended beyond data only, to include the full life-cycle of a first-principles simulation, including the workflows that generate the data, the codes that execute them, and the interactive tools used to analyze the results^7^. Below, we map each FAIR dimension onto concrete features that are already available in the QE app or planned for the near future, underscoring the added value for both end-users and developers.

In terms of findability, every calculation launched from the QE app is automatically stored in the AiiDA provenance graph. Users can export the complete graph—including inputs, outputs and metadata—as a single AiiDA archive file. These archives can be deposited in the *Materials Cloud Archive*^58^, which offers persistent DOIs and a rich web interface that lets third parties browse the full data lineage and inspect input and output files. At the workflow layer, AiiDA’s entry-point mechanism registers every workflow, parser and calculation plugin with a unique name and provides a plugin registry for plugin developers to register their workflows. At the plugin layer, the app provides a *Plugin management* page, where users can discover and install additional plugins from the community.

Accessibility is achieved through flexible deployment modes. The QE app is available in both cloud-based and local configurations, lowering the entry barrier for non-specialists. In the cloud, it can be accessed directly through a web browser without installation or setup. The cloud deployment is managed by Kubernetes and JupyterHub, which handle authentication and authorization via standard OAuth2 protocols, ensuring secure and scalable access. To provide an immediate hands-on experience, we also offer a publicly accessible demonstration instance at https://demo.aiidalab.io. For local use, the app can be launched using the aiidalab-launch utility, which sets up a pre-configured, containerized environment. These deployment options (see App accessibility section) ensure flexibility and broad accessibility across user environments.

Interoperability is a key strength of the app’s architecture. While the app currently supports only QE, its architecture is designed to be interoperable by serving as a generic template that can be adapted to other DFT codes. The core components are code-agnostic: the AiiDA-powered backend, the AiiDAlab-powered frontend, the IPO model, and the modular wizard UI. Adapting this framework for a new code, therefore, does not require re-engineering the entire application. Instead, a developer can focus on implementing code-specific input schema and graphical interface extensions, while the overall architecture remains unchanged. By exploiting the common workflow interfaces for over ten DFT codes that have been defined and implemented in AiiDA^6,31^, developers can reuse the app’s structure to build similar applications for other quantum engines. For example, we are currently exploring the adaptation of this framework to the CP2K code by integrating the independently developed aiidalab-empa-surfaces app^109^ into the same structural architecture used in the app. The app’s architecture is also serving as a model for a DFT-code-agnostic GUI developed as part of the PREMISE project of the ETH Board (https://ord-premise.org/). Furthermore, the interactive widgets and results analysis tools integrated into the app have proven invaluable not only for non-specialists but also for seasoned computational materials scientists. To maximize their utility, we plan to further modularize these tools, allowing users to employ them outside of the app’s main interface, such as in a standard Jupyter notebook. These tools can also be utilized on other platforms, like the Materials Cloud website^58^ and the OSSCAR platform^110,111^.

Reusability and reproducibility are ensured through open-source licensing and the integration of AiiDA’s provenance tracking. All components of the QE app, including the main interface, the underlying AiiDA plugins, and the analysis widgets, are released under permissive open-source licenses and hosted on public GitHub repositories (see the **Code Availability** section), maximizing reusability and transparency. By using AiiDA for workflow management, the app ensures that all inputs, processes, and outputs are automatically stored in a structured, queryable database. When a calculation is restarted (e.g., to resolve SCF convergence issues), the updated settings are also recorded, preserving the full decision-making process. Even publication-quality figures generated via the interactive plotting widgets are fully reproducible with a single click, as the complete widget state (e.g., axis ranges, color maps, structure viewer settings) is stored alongside the corresponding AiiDA node. While reproducibility goes beyond the original FAIR acronym, our design makes it a practical reality and aligns with the provenance-focused spirit of the FAIR guidelines. Furthermore, the app’s plugin architecture ensures extensibility and modularity, allowing users and developers to share and reuse specialized workflows for diverse material properties and advanced analytical techniques, thus facilitating broader community collaboration.

Apart from FAIR, the development of the QE app also underscores the importance of user-friendly interfaces. These interfaces lower entry barriers for non-specialists and accelerate the onboarding process. For example, the in-app guide system, which overlays context-specific information and “click-here-next” instructions on top of any panel, trains the user in place, avoiding the need for external manuals (see Supplementary Fig. S14). Ongoing development efforts aimed at decoupling the UI from the AiiDA backend via modern UI frameworks and dedicated REST APIs will significantly enhance scalability, multi-user capabilities, and collaborative potential. Such architectural evolution will enable more efficient use of computational resources, broader access, and robust support for collaborative research initiatives, further solidifying the app as a cornerstone platform in the FAIR simulation landscape.

The QE app represents a significant advancement in making DFT simulations more accessible to the broader communities by offering turn-key solutions that simplify the complexities of computational materials science. Through its intuitive GUI, the app guides users from structure selection to results analysis, reducing barriers for non-specialists. While not intended to replace proper training, it offers a scientifically sound entry point supported by initiatives, such as our dedicated training sessions^112^, helping non-specialists learn through guided practice while gradually building expertise. Its modular, plugin-based architecture supports diverse materials science workflows and allows for the rapid integration of new functionalities, keeping the app up to date with ongoing developments in computational materials science. In conclusion, the QE app lowers the barrier to advanced DFT simulations, enabling a broader range of researchers to adopt computational tools and workflows, accelerating collaboration and driving forward materials research.

## Methods

### Technical details

Given the complexity of the QE app’s UI and underlying logic, careful architectural choices are essential to ensure long-term maintainability and extensibility. To this end, the app adopts the Model-View-Controller^113,114^ (MVC) design pattern, which provides a clear separation between the UI, the underlying data models, and the computational logic. This structure enhances scalability, simplifies testing, and improves responsiveness by enabling lazy-loading techniques. The details of the MVC implementation, including the use of the Observer and Mediator supporting design patterns via the traitlets package, and the model network architecture, are provided in the **Model-View-Controller** section of the Supplementary Information.

### Computational details

All DFT calculations in this work are performed using the default settings of the aiida-quantumespresso balanced protocols, as defined in ref. ^62^. The SSSP v1.3.0 efficiency pseudopotential family^63,115^ is used throughout. For the phonon simulations of BaZrS_3_ shown in Fig. 4, a 2 × 2 × 2 supercell is employed to compute the force constants, which are necessary to obtain the phonon dispersion and related vibrational properties.

## Supplementary information

Supplementary Information

## Data Availability

The QE app is available as an open-source project and can be accessed from its GitHub repository: https://github.com/aiidalab/aiidalab-qe. The aiida-quantumespresso plugin and its associated workflows are hosted at https://github.com/aiidateam/aiida-quantumespresso. The AiiDAlab platform, which serves as the foundation for these applications, is maintained under the AiiDAlab GitHub organization: https://github.com/aiidalab The aiidalab-launch tool is hosted at https://github.com/aiidalab/aiidalab-launch. Additionally, QE app plugins for different functionalities are available: • aiidalab-qe-vibroscopy: https://github.com/aiidalab/aiidalab-qe-vibroscopy • aiidalab-qe-muon: https://github.com/aiidalab/aiidalab-qe-muon • aiidalab-qe-hp: https://github.com/aiidalab/aiidalab-qe-hp • aiidalab-qe-wannier90: https://github.com/aiidalab/aiidalab-qe-wannier90 • aiida-qe-xspec: https://github.com/aiidaplugins/aiida-qe-xspec • aiidalab-qe-pp: https://github.com/AndresOrtegaGuerrero/aiidalab-qe-pp • aiida-bader: https://github.com/superstar54/aiida-bader All software mentioned in this paper is open-source and freely available to the community.
