# Supplementary material for: Making atomistic materials calculations accessible with the AiiDAlab Quantum ESPRESSO app
Source: NPJ Comput Mater. 2026 Feb 3;12(1):72. doi: 10.1038/s41524-025-01936-4 (PMC12875877; doi:10.1038/s41524-025-01936-4)
Supplement: Supplementary file 1 — Supplementary Information [file 41524_2025_1936_MOESM1_ESM.pdf]

Supporting Information: Making atomistic materials calculations accessible with AiiDAlab

Xing Wang[1,2,†*], Edan Bainglass[1,2,†], Miki Bonacci[1,2,†], Andres Ortega-Guerrero[3,†], Lorenzo Bastonero[4], Marnik Bercx[1,2], Pietro Bonfà[5,6], Roberto De Renzi[7], Dou Du[8], Peter N. O. Gillespie[6], Michael A. Hernández-Bertrán[5,6], Daniel Hollas[9], Sebastiaan P. Huber[8], Elisa Molinari[5,6], Ifeanyi J. Onuorah[7], Nataliya Paulish[1,2], Deborah Prezzi[6], Junfeng Qiao[8], Timo Reents[1,2], Christopher J. Sewell[8], Iurii Timrov[1,2], Aliaksandr V. Yakutovich[3], Jusong Yu[1,2], Nicola Marzari[1,2,4,8], Carlo A. Pignedoli[3,*], and Giovanni Pizzi[1,2,8,*]

[1]PSI Center for Scientific Computing, Theory and Data, 5232 Villigen PSI, Switzerland
[2]National Centre for Computational Design and Discovery of Novel Materials (MARVEL), 5232 Villigen PSI, Switzerland
[3]nanotech@surfaces Laboratory, Empa-Swiss Federal Laboratories for Materials Science and Technology, 8600 Dübendorf, Switzerland
[4]U Bremen Excellence Chair, Bremen Centre for Computational Materials Science, and MAPEX Center for Materials and Processes, University of Bremen, 28359 Bremen, Germany
[5]Dipartimento di Scienze Fisiche, Informatiche, Matematiche (FIM), Università di Modena e Reggio Emilia, 41125 Modena, Italy
[6]Nanoscience Institute, National Research Council (CNR-NANO), 41125 Modena, Italy
[7]Department of Physics and Earth Sciences, University of Parma, 43124 Parma, Italy
[8]Theory and Simulation of Materials (THEOS), École Polytechnique Fédérale de Lausanne, 1015 Lausanne, Switzerland
[9]Center for Computational Chemistry, School of Chemistry, University of Bristol, BS8 1TS Bristol, UK
[†]these authors contributed equally to this work
[*]Corresponding authors: Xing Wang (xing.wang@psi.ch), Carlo A. Pignedoli (carlo.pignedoli@empa.ch), Giovanni Pizzi (giovanni.pizzi@psi.ch)

Input-Process-Output model

As introduced in the main text, the Input-Process-Output (IPO) model provides the architectural pattern for the QE app's unified interface. This section provides a detailed breakdown of its implementation at both the backend (workflow) and frontend (GUI) levels.

On the workflow side, as shown in Fig. S1, we provide a top-level AiiDA WorkChain that embodies the IPO logic:

1. **Inputs**: provide a common set of input parameters used by all property-specific sub-workflows. At the same time, the full inputs of these sub-workflows are exposed and can be overridden by advanced users.

2. **Process**: execute the appropriate sub-processes (e.g., relaxation, band structure, phonons) based on the selection;

3. **Outputs**: exposing outputs from sub-processes for easy access and post-processing.

This design provides both standardization and flexibility: while a consistent high-level interface exists for all property types, advanced users can override any property-specific parameter by overriding the parameters generated from the protocol.

In the GUI implementation, the IPO structure of the backend workflow is naturally mirrored. The GUI is organized into three main phases corresponding to the Input, Process and Output. For convenience of the users, the Input phase is further divided into a structure selection step, a parameter setting step, and a submission step. The Process phase displays a process tree that monitors the progress of all calculations. The Output phase displays the results and analysis.

These steps are implemented using a wizard UI that guides users through the simulation process. Similar to the workflow layer, the GUI provides both general (e.g., protocol, electronic type, magnetic type) and property-specific settings pages, ensuring accessibility for non-experts and flexibility for advanced users.

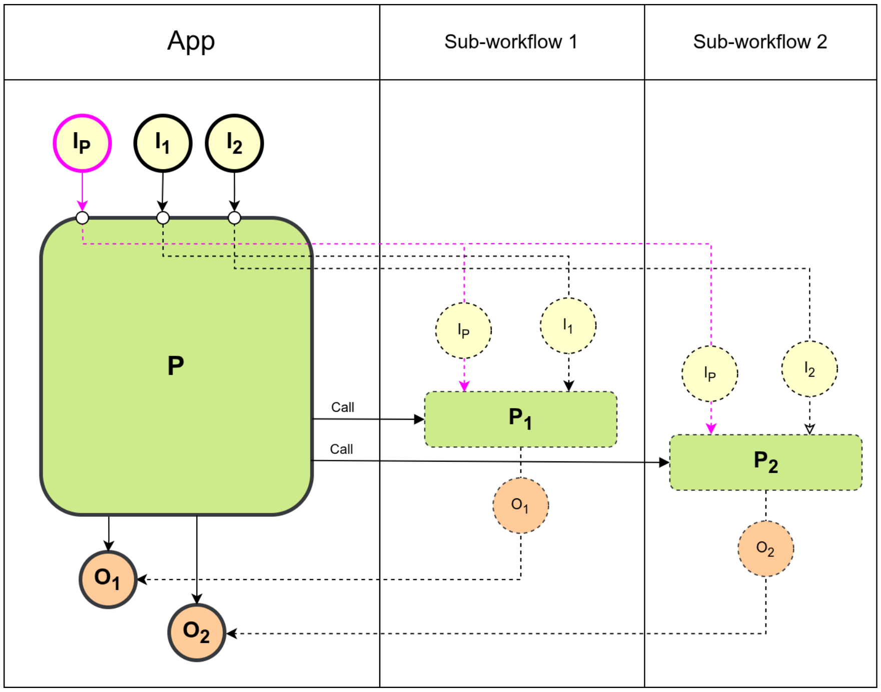

Figure S1. Schematic representation of the workflow structure in the QE app following the IPO model. The main app workflow (P) collects a common set of basic input parameters (I_P, e.g., protocol, electronic type [if metallic or not], ...) shared by all property-specific sub-workflows. These inputs, together with optional property-specific inputs (I_1, I_2), are passed to the corresponding sub-workflows (P_1, P_2) responsible for computing different material properties. Each sub-workflow produces its own outputs (O_1, O_2) while exposing them back to the main app for post-processing and visualization. The dashed connections indicate how inputs and outputs are propagated between the app and its sub-workflows, ensuring both a consistent structure and full flexibility for property-specific customization.

Workflow parameter settings panels

Although each workflow already encapsulates a wealth of expert knowledge, DFT simulations often involve fine-tuning numerous input numerical parameters, such as basis set size (e.g., wavefunction and charge-density cutoffs in the case of plane-wave methods as is the case for QUANTUM ESPRESSO), **k**-point sampling, choice of type and magnitude of the smearing of the electronic states, or convergence thresholds. The workflow configuration step aims to balance abstraction of complexities involved in calculation setup with flexibility of customizing calculation parameters often required by advanced users. To do so, the app splits the step into basic, advanced, and property-specific panels, each covering one aspect of this goal. Below, we describe briefly the features and purpose of each panel.

Basic settings - top-level controls and calculation protocols

To streamline workflow setup, we provide in this panel top-level controls to specify general considerations for the system (metallic/insulating, magnetic, spin-orbit coupling - see Fig. S2). In addition, to abstract the complexities of configuring DFT calculations, we implement a protocol system which pre-configures a curated set of computational settings for different levels of accuracy and computational cost (Table S1). We provide three predefined protocols, each tailored to a specific use case and benchmarked in Ref. 1:

1. **Fast**: Optimized for speed, this protocol is intended only for testing purposes or quick, preliminary investigations where computational efficiency is the priority.

2. **Balanced**: An intermediate option that is typically recommended as the default, as it provides a balanced trade-off between computational cost and numerical precision.

3. **Stringent**: Designed for highly precise results, this protocol employs more stringent settings, suitable for cases where precision is paramount at the expense of a higher computational cost.

Other than the parameters shown in Table S1, the protocols also affect other workflow-level settings, such as the volume convergence used in structural relaxations. In addition, the "Stringent" protocol also selects a pseudopotential with higher plane-wave and charge-density cutoffs. All defaults can be overridden by users.

These protocols have been developed and tested for a large database of structures. By automatically setting key parameters like **k**-point meshes and convergence thresholds, the protocol system relieves users of the need for manual configuration, thereby reducing the potential for misconfiguration and improving overall workflow reliability.

Table S1. Protocol-dependent default parameters for self-consistent field (SCF) calculations.

**Protocol**	**Thresholds**			**Smearing width (Ry)**	**k-spacing (Å^{-1})**
	E_{SCF} (Ry/atom)	E_{ionic} (Ry/atom)	F_{ionic} (Ry/bohr)		
Fast	$4 \cdot 10^{-10}$	$1 \cdot 10^{-4}$	$4 \cdot 10^{-3}$	0.0275	0.3
Balanced	$2 \cdot 10^{-10}$	$1 \cdot 10^{-5}$	$4 \cdot 10^{-4}$	0.02	0.15
Stringent	$1 \cdot 10^{-10}$	$5 \cdot 10^{-6}$	$5 \cdot 10^{-5}$	0.0125	0.1

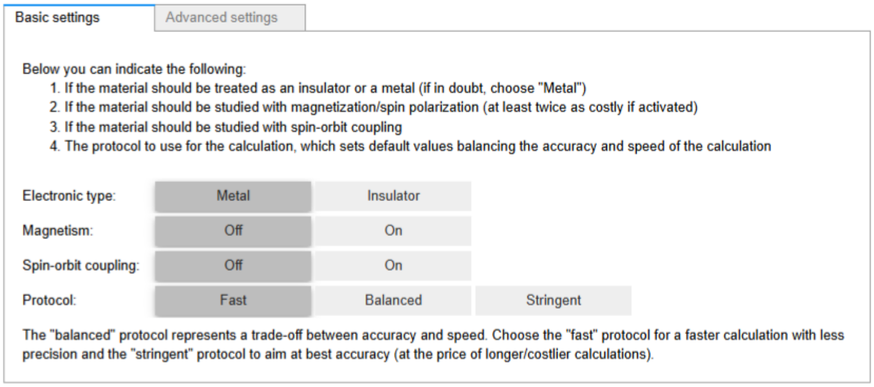

Figure S2. The basic parameter-settings panel providing users with top-level controls to streamline the process of configuring the calculation. These include defining the electronic type (metallic or insulating), switching on magnetism (spin polarization) and spin-orbit coupling, and choosing a calculation protocol, pre-configuring a set of calculation parameters balancing calculation speed and accuracy (see Table S1).

Advanced settings - flexibility and tunability

In this panel, we provide controls for users more familiar with the underlying code and theory to override protocol-specific defaults (convergence criteria, Brillouin zone **k**-point sampling, smearing, cutoffs) and customize system/site properties (magnetization, Hubbard parameters) and selected pseudopotentials (Fig. S3). The panel includes a reset button to re-apply protocol-dependent defaults. We note that for Hubbard parameters, though a plugin is available to compute Hubbard U+V (see **Hubbard-parameter calculations** section below), we presently support in the app the use of Hubbard U only. Development is ongoing to introduce controls for providing also Hubbard V.

Plugin-specific settings

Additional settings panels are provided for each selected plugin property, in which plugin developers provide users with a set of controls for tuning the calculation(s) involved in the plugin's workflows. Further details on plugin settings panels is given in the **Plugins** section below.

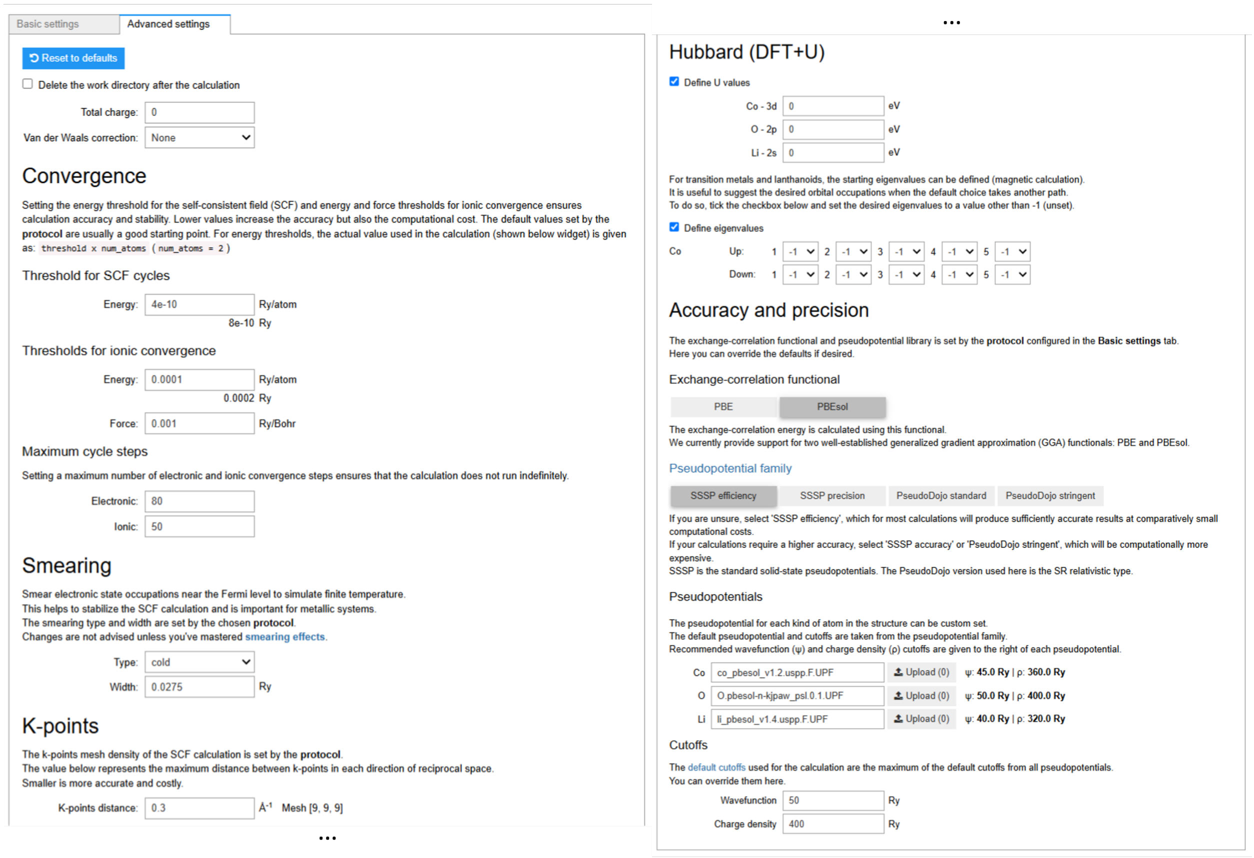

Figure S3. The advanced settings panel providing users with a wide range of controls to set convergence criteria, smearing, the k-point grid, Hubbard U parameters, pseudopotentials, and wavefunction and charge density cutoffs. If magnetism is switched on (spin-polarized calculation), additional controls are displayed to parameterize the magnetic calculation (not shown).

Plugins

The QE app supports running multiple properties (bands, PDOS, etc.) calculations in one app. The individual properties can be developed and seamlessly integrated into the app as plugins. This integration is made possible since for most properties, the following conditions are met:

- the configuration for a property calculation has its settings unrelated to other properties;
- the sub-workflow of the properties can be run independently;
- the analysis of the results of the properties is independent.

Each plugin is responsible for one property calculation. A plugin typically registers new panels (setting, result) and workflows (AiiDA WorkChains) to extend the functionality of the app. The plugin design makes the QE app modularized and pluggable. Consequently, developers have the flexibility to manage their plugins in a distinct folder within the QE app codebase, or they may choose to maintain it as an independent package.

Muon spectroscopy

Muon spin rotation and relaxation spectroscopy is a fundamental experimental technique used to probe local magnetism, superconductivity and charge ordering mechanisms of materials[2]. The experimental setup is briefly summarized in the following. A positive muon with kinetic energy $E_k > 4$ MeV is implanted in a sample, losing gradually energy up to thermalization in a given (unknown) interstitial site in the crystal. The muon then interacts with the local magnetic

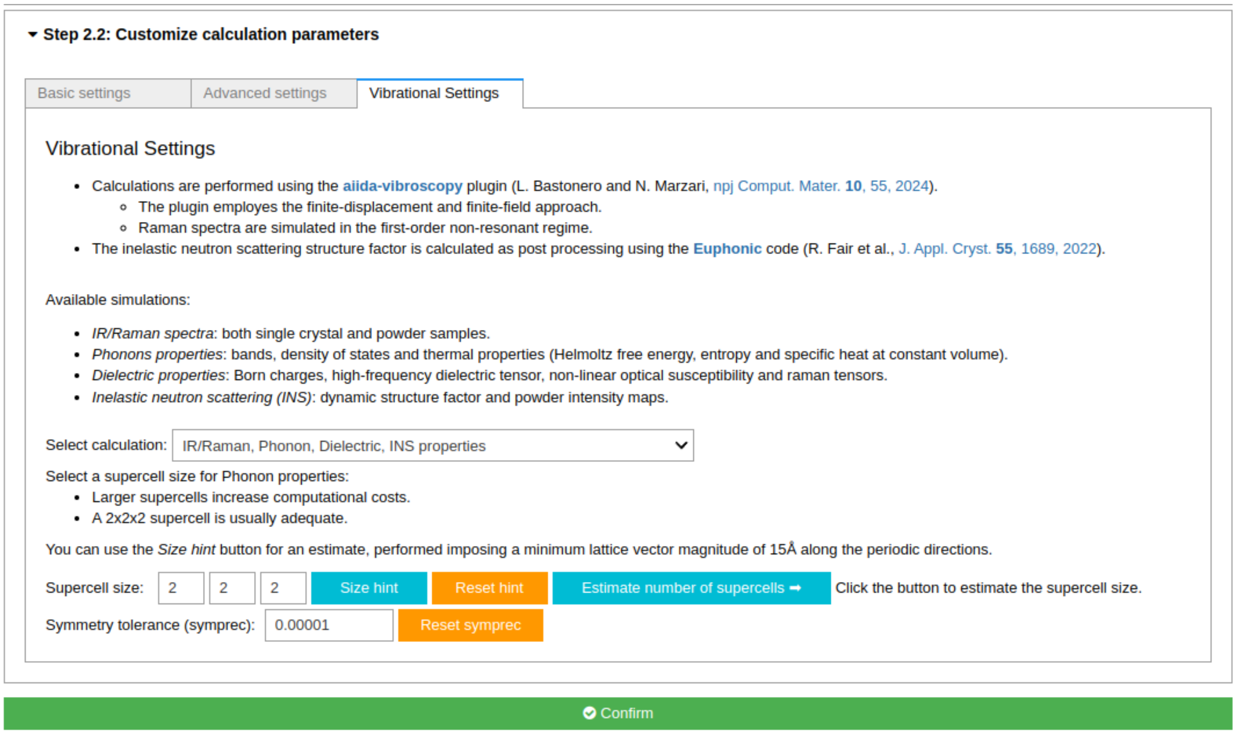

Figure S4. The configurations settings panel for the aiidalab-qe-vibroscopy plugin.

environment and, consequently, its spin polarization $P(t)$ undergoes a precession. The time evolution of $P(t)$ is the main experimental outcome. To correctly interpret the signal, it is fundamental to determine the muon resting sites in the material, as well as its local microscopic interactions. Experimentally, this is far from trivial and first-principles methods are therefore almost essential, as they can predict with great accuracy both the resting sites and the related local fields as felt by the muons. In particular, the approach called DFT+μ[3,4] models the muon as an infinitely dilute impurity in the system and computes its property by means of DFT. This approach requires running a large number of supercell calculations, each with the muon placed in a different trial position. Once the muon resting sites are determined, it is possible to obtain the time evolution of the muon spin polarization originating from its interaction with nuclear magnetic dipoles in standard experimental conditions (i.e., when thermal energy is much larger than nuclear interactions), as e.g. computed by the UNDI package[5].

In the QE app, DFT+μ workflows are accessible by installing the `aiidalab-qe-muon` plugin[6]. The plugin implements the AiiDA `ImplantMuonWorkChain` workflow for the search of muon resting sites within a given material. The `ImplantMuonWorkChain` manages various sub-WorkChains dedicated to muon spectroscopy, implemented in the `aiida-muon` and `aiida-impuritysupercellconv` AiiDA plugins[7–10]. The former specializes in locating resting sites and magnetic interactions by means of DFT+μ, while the latter is designed to automatically determine the appropriate supercell size for muon simulations (or, in general, infinitely dilute H impurities). A dedicated *Settings* panel allows users to configure and customize muon-related DFT+μ parameters, namely: supercell size, muon charge state, density of muon trial positions, inclusion of Hubbard corrections and spin polarization. If only a fast estimation of the needed supercell size is desired, this can instead be obtained live through a designated *Hint* button, bypassing explicit submissions of AiiDA workflows and utilizing instead heuristic estimates. Furthermore, it is possible to enable the post-processing calculation of the muon polarization (via the `UNDI` package) for predefined combinations of external magnetic field values and sample orientations. A results panel collects and visually summarizes all simulations outputs, as illustrated in Fig. S5. Specifically, all detected candidate muon resting sites are listed in a table alongside their associated relative total energy, with the lowest-energy one serving as the reference. A plot of the hosting lattice distortions is presented as useful hint to identify self-trapped muons. The sites are also visualized in a 3D viewer and users can seamlessly switch between two viewing modes: show each individual site within the corresponding supercell, or wrap all identified sites within the unit cell. If requested during the configuration phase, a prediction of the

depolarization function for each site is also provided. Finally, the supercells for each of the detected resting sites and the associated data shown in the results table can be easily downloaded for further post-processing.

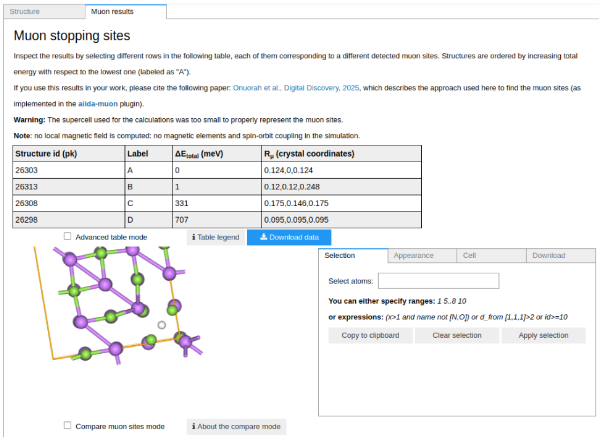

Figure S5. Results panel for the muon spectroscopy plugin, in the case of lithium fluoride LiF. The panel contains an informative table on the candidate muon sites and their relative total energy, as well as the position in the supercell, and possibly magnetic interactions (which are not shown in this case, as the system was not magnetic). Moreover, the supercell with the stopping muon site is presented. Users can interactively inspect it (for example computing the distance between the muon and the neighboring hosting atoms).

X-ray core-level spectroscopies

In the QE app, we provide additional plugins designed to automate the computation of core-level spectroscopies, covering X-ray photoelectron spectroscopy (XPS) and X-ray absorption spectroscopy (XAS) calculations.

XPS, a technique based on the photoelectric effect, stands as an important technique in modern surface and materials science. It finds extensive applications across diverse domains such as semiconductors, energy, environmental science, catalysis, and corrosion[11]. The so-called Delta Kohn-Sham (ΔKS) approach[12,13] is routinely used to rationalize XPS data. Starting from core-hole pseudopotentials, it allows one to compute the XPS binding energies as total energy differences of the systems with and without core hole for each given species in the target system[14]. We leverage the automated robust workflow to compute XPS spectra implemented in the `aiida-qe-xpsec`[15] package, which uses the ΔKS approach and pseudopotential-based total energy calculations, and we integrate it inside the QE app as a plugin, exposing it via a user-friendly interface. A dedicated configuration panel allows users to choose the system type (molecule or crystal), core-hole pseudopotential group, core levels, and target atoms for analysis (see Fig. S6). In the result panel, users can interactively adjust the spectral broadening and intensity parameters and view site-resolved shifts in a table that links directly to the 3D structural viewer (see Fig. S7). One can also upload reference experimental XPS data to compare with the calculated results.

Similarly, XAS has served for many decades an important role in materials science as a means to gain information about the local chemical environments present in a given material[16]. Theoretical approaches to analysing both the X-ray absorption near edge (XANES), up to 50 eV above the absorption onset, and the extended X-ray fine-structure (EXAFS) region beyond the near edge have been well established in the literature. Methods for computing XANES may vary by theoretical framework (for an overview see e.g. Refs. 17, 18) and computational approach, including real-space approaches based on wavefunctions[19,20], multiple-scattering[21] as well as reciprocal-space, band-structure methods[22,23] within DFT. For the calculation of K-edge XANES, for which single-particle methods are known to describe with good accuracy[17], we provide a plugin exposing a simple and user-friendly interface to the underlying robust workflows implemented in the `aiida-qe-xspec`[15] package. These workflows use the `xspectra.x`[24,25] code of QUANTUM ESPRESSO, adopting the projector augmented-wave (PAW) method in a reciprocal-space pseudopotential scheme, where the XANES spectrum is computed from a cross-section between a given core level (initial state) and the virtual (unoccupied) states (final states) of the material in the presence of a localized core-hole. The plugin can compute XANES spectra for any given solid exploiting crystal symmetry analysis to identify equivalent absorbing atom sites (if any). The choice of different core-hole treatments is offered to the user, i.e., full, half and excited core-hole[12,26], together with other options important to completely resolve the XANES spectrum of the material. Moreover, in the app

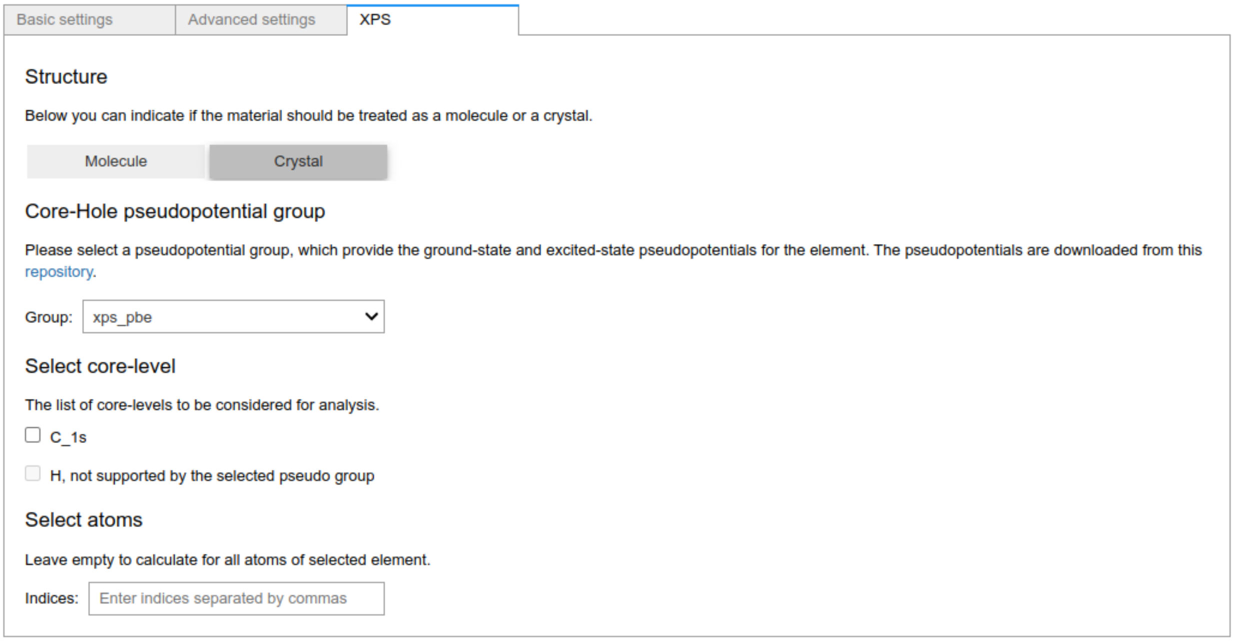

Figure S6. Configuration panel for the XPS plugin. Users can define whether the system is a molecule or a crystal, select the pseudopotential group (including core-hole versions), choose specific core levels for analysis (e.g., C 1s), and optionally limit the calculation to specific atomic indices of the selected element.

we provide a set of well-tested core-hole pseudopotentials for several common elements (e.g. C, O, F). As an example, Figure S8 shows how the total K-edge for a selected element can be inspected in the XAS results panel, in addition to the K-edge XANES of sub-components belonging to symmetrically-inequivalent absorbing atoms. The plugin also features a set of post-processing tools to apply core-hole lifetime broadening effects to the computed spectrum based on the formulation of Bunau *et al.*[25] which closely matches the typical broadening seen in experimental spectra. The resulting spectra may then be downloaded for further analysis.

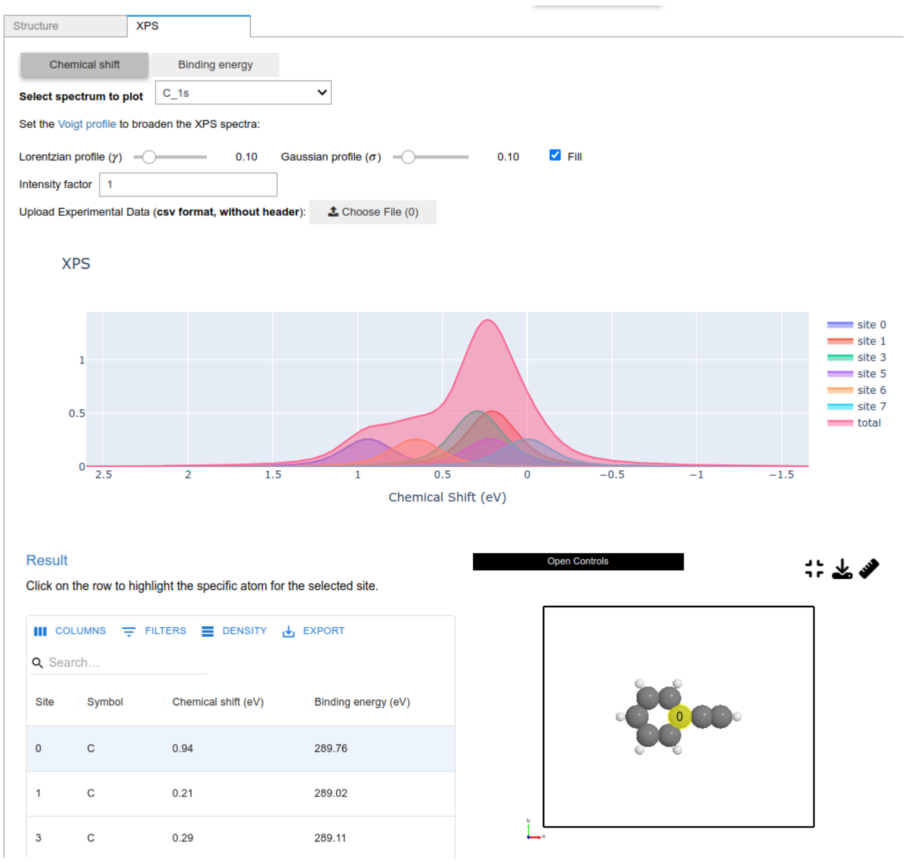

Figure S7. Results panel for the XPS plugin, showing the C 1s core level shift of phenylacetylene in gas phase. Users can interactively select the element (e.g., C), toggle between chemical shift and binding energy, and adjust the Voigt profile parameters (Lorentzian and Gaussian broadening) to simulate the experimental broadening effects. A summary table lists the chemical shift and binding energy values for each atomic site, with the option to highlight the corresponding atom in the 3D molecular structure upon selection.

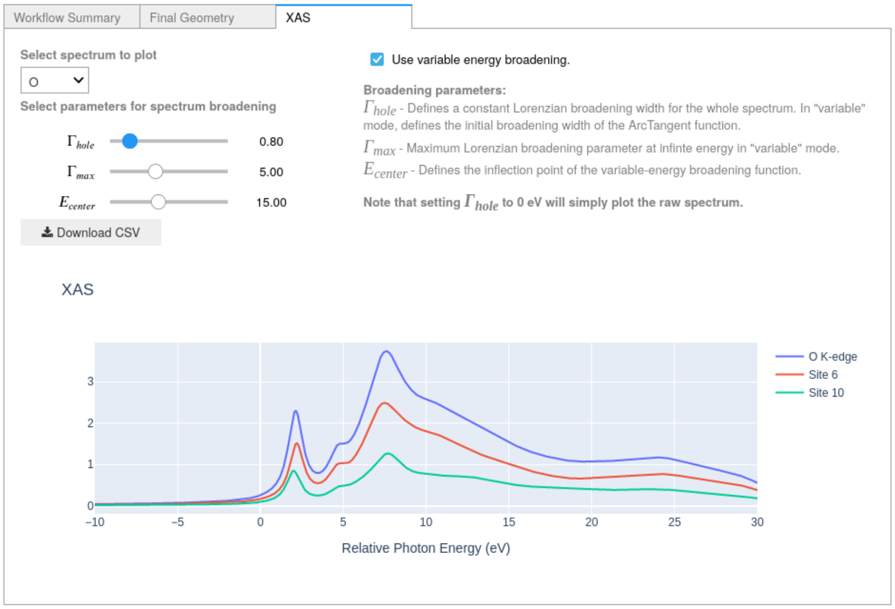

Figure S8. Results panel for the XAS plugin, showing the example of the O K-edge XANES of lithium carbonate (Li_2CO_3). Shown in the plot window are the XANES of the two symmetrically-inequivalent O sites of Li_2CO_3 (red/green) in addition to the full O K-edge XANES (blue). Users can interactively vary the spectrum broadening parameters (upper left of panel), switch between constant and variable broadening types (checkbox, top center of panel), and export spectra data in csv format (download button, center left of panel). Other edges calculated during the same workflow run may be selected using the dropdown menu in the top left of the panel.

Wannier-function calculations

The concept of maximally-localized Wannier functions (MLWFs) is important for understanding, interpreting and efficiently computing a wide range of electronic structure properties in condensed matter, from chemical bonding analyses to constructing tight-binding Hamiltonians for transport and topological characterizations[27–29]. Leveraging the open-source Wannier90 code[30], the plugin for Wannier functions (WFs) seamlessly orchestrates two main stages of the workflow (implemented in the `aiida-wannier90-workflows` package[31,32]) in an automated and user-friendly environment. A workflow is first launched to compute the SCF charge density and the corresponding band structure using DFT via QUANTUM ESPRESSO. The SCF density and bands are then used as inputs to a `Wannier90OptimizeWorkChain`. Within the plugin configuration panel, users can select among various approaches including the selected columns of the density matrix (SCDM) approach[33], and projectability disentanglement (PDWF method)[31], as well as choose a "frozen" energy window method to tailor the localization procedure to the system band structure[31]. An additional checkbox triggers the optional calculation of the Wannier functions in real space. In order to generate the Fermi surface and compute de Haas–van Alphen oscillation frequencies, the user needs to activate the respective checkboxes. The Fermi surface is computed at the last step of the Wannierization procedure, using the Wannier90 code. de Haas–van Alphen oscillation frequencies are computed using the open-source SKEAF code[34].

Once the calculations are finished, the plugin presents a comprehensive result page. It superimposes the DFT and Wannier-interpolated band structures in an interactive plot for comparison, as shown in Fig. S9, highlighting their band distance (see Ref. 31 for the definition). Below, a summary table displays key outputs of the Wannier90 code including the number of Wannier functions, the final WF spreads (Ω_{tot}), their individual components ($\Omega_D, \Omega_I, \Omega_{\mathrm{OD}}$) and the band distance. Additional panels offer line plots for monitoring the iterative convergence of the localization spreads, a detailed table of Wannier centers and spreads (both initial and final), and a 3D viewer for the structure. By selecting any row in the table, users can simultaneously visualize the isosurfaces of the selected Wannier function and highlight the corresponding atoms that are close to the Wannier center in the 3D structure viewer, as shown in Fig. S9. The interactive 3D visualization is powered by `weas-widget`[35], which is also utilized in the post-processing plugin (see next section on Post-processing plots). An additional 2D plot visualizes the de Haas–van Alphen oscillation frequencies. The tight-binding model as well as the Fermi surface files are available for download in the "Downloads" panel.

Overall, the Wannier function plugin within the QE app streamlines the entire workflow, from plane-wave band structure computations to MLWF localization, offering an intuitive, graphical interface for both setup and post-processing.

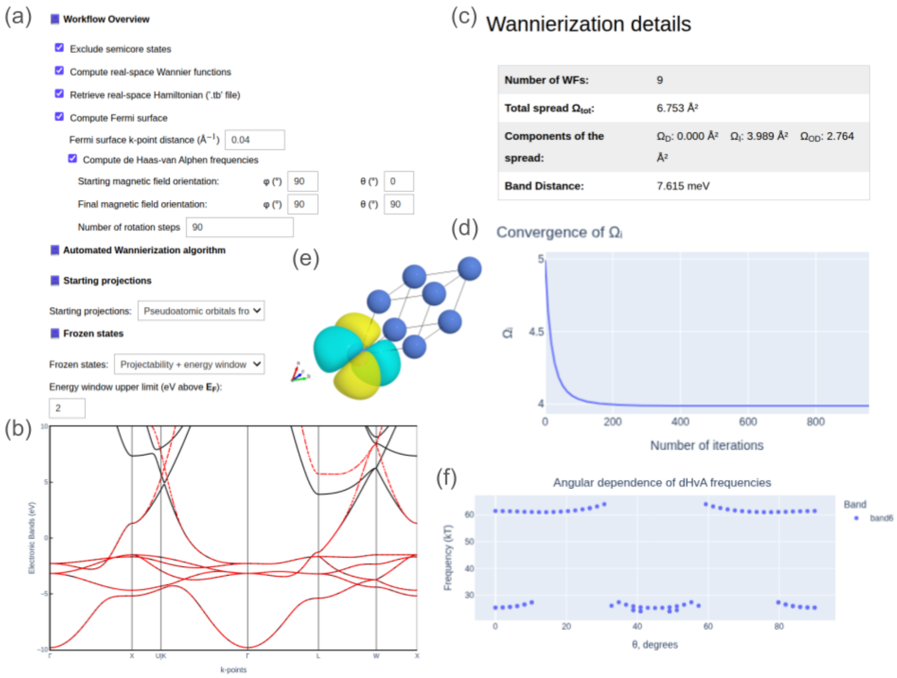

Figure S9. Panels for the Wannier function plugin in the QE app, illustrated in the case of a copper crystal. (a) Configuration panel used to set up the Wannierization workflow, including projection scheme, energy window, and optional generation of real-space Wannier functions, Fermi surface, and de Haas–van Alphen oscillation frequencies. (b) Comparison between the DFT band structure (black) and Wannier-interpolated bands (red), demonstrating the quality of interpolation. (c) Summary of Wannierization results showing the number of Wannier functions, spread components, and band distance between DFT and Wannier bands. (d) Convergence behavior of the Wannier function spread components. (e) 3D visualization of a selected maximally localized Wannier function, with its isosurface and nearby atoms highlighted in the structure viewer. (f) Angular dependence of de Haas–van Alphen oscillation frequencies.

Hubbard-parameter calculations

DFT with local or semilocal exchange-correlation functionals—such as the local-density approximation (LDA) and generalized-gradient approximation (GGA)—is a cornerstone of materials science. However, it suffers from significant self-interaction errors in systems with partially filled d- and/or f-shells, such as transition-metal and rare-earth compounds. To mitigate these errors, various advanced approaches have been developed, among which Hubbard-corrected DFT (DFT+U[36–38] and DFT+U+V[39]) stands out for its balance between accuracy and computational efficiency. A key challenge of this approach is the determination of the Hubbard U and V parameters. While these are often tuned empirically using experimental data, first-principles methods offer a more reliable alternative. Among them, the supercell-based linear-response method[40] and its reformulation using DFPT in primitive cells[41,42] have proven to be both accurate and robust for computing Hubbard parameters.

The plugin provides an interface to the `aiida-hubbard` AiiDA plugin[43,44], based on the `hp.x` code[45] of QUANTUM ESPRESSO that uses DFPT to compute the on-site U and inter-site Hubbard parameters V in a self-consistent, fully *ab initio* manner, ensuring that both the electronic ground state and the Hubbard corrections converge in tandem. The plugin provides an intuitive panel for defining calculation modes, specifying which atoms (for U) or pairs of atoms (for V) require Hubbard corrections, and setting convergence tolerances (see Fig. S10a). Users begin by selecting either the one-shot or self-consistent method from a dropdown menu. Specifically, the one-shot mode performs a single pass, fixing the geometry and extracting U (and optionally V) parameters for the specified orbitals,

while the self-consistent mode applies an iterative routine, wherein the structure can be re-optimized at each step. The Hubbard parameters are updated until convergence criteria on U and V (and optionally the structure) are satisfied. Next, users select whether to include only on-site U or both on-site U and inter-site V corrections. A scrollable table automatically lists each atomic kind in the structure, allowing one to toggle corrections for specific sites and input an initial guess for U. For inter-site interactions, pairs of atomic kinds are similarly exposed, with fields to activate and set an initial guess for V. Finally, advanced settings such as parallelization over individual atoms and **q** points for DPFT calculations can be enabled through checkboxes. These options leverage the inherent parallelization capabilities of the `hp.x` code[45] to significantly reduce walltime, particularly for large simulation cells or complex materials. Once the calculation is complete, the DFT+U+V plugin presents the Hubbard parameters in an interactive table alongside the atomic structure (see Fig. S10b). This feature allows users to directly correlate computed U and V values with specific atoms or atom pairs selected in the table. The computed values can then be used as inputs for subsequent DFT+U+V calculations (e.g., phonons, IR/Raman, XAS, XPS, ...) or other advanced electronic structure analyses (see **Advanced settings – flexibility and tunability** above for how to manually set Hubbard parameters in the app).

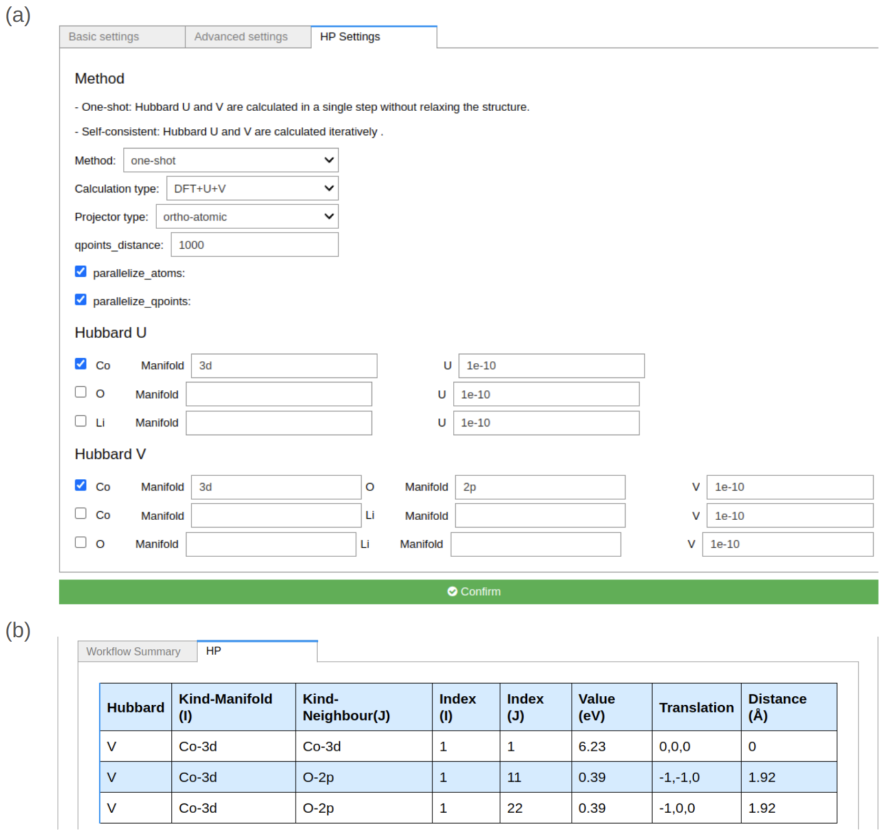

Figure S10. User interface of the plugin for calculating Hubbard parameters. (a) Setup panel for defining the Hubbard parameter calculation: users can select between one-shot and self-consistent modes, choose the calculation type (DFT+U or DFT+U+V), and configure which atomic sites or atom pairs should receive on-site (U) or inter-site (V) corrections. Additional fields allow setting initial guesses and enabling parallelization over atoms and **q** points. (b) Results panel showing computed Hubbard parameters for selected atom pairs in a $LiCoO_2$ structure.

Post-processing plugin

The QE app supports an external plugin to run post-processing calculations using `pp.x`, based on wavefunction files produced from previously executed PDOS or band structure calculations. To use the plugin, users must select a structure associated with a completed PDOS or Bands calculation. Fig. S11 shows the configuration panel of the plugin. It lists all relevant `PwCalculation` entries linked to the selected structure and provides essential metadata, such as whether LSDA or spin-orbit coupling was used. This information is required to determine the appropriate `pp.x` post-processing

logic. Additionally, the plugin displays the AiiDA computer on which the original calculation was performed. This is critical, as the post-processing must be executed on the same machine where the original calculation was run in order to access the needed files. Once the selected post-processing calculations are completed, the results will be displayed in a separate tab featuring a 3D plot, as shown in Fig. S12.

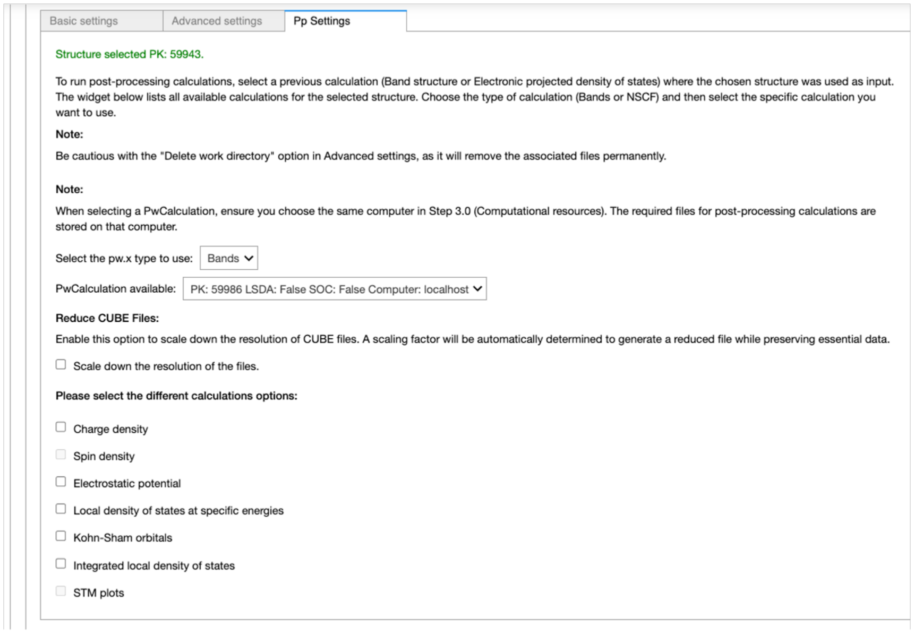

Figure S11. The configurations settings panel for the aiidalab-qe-pp plugin.

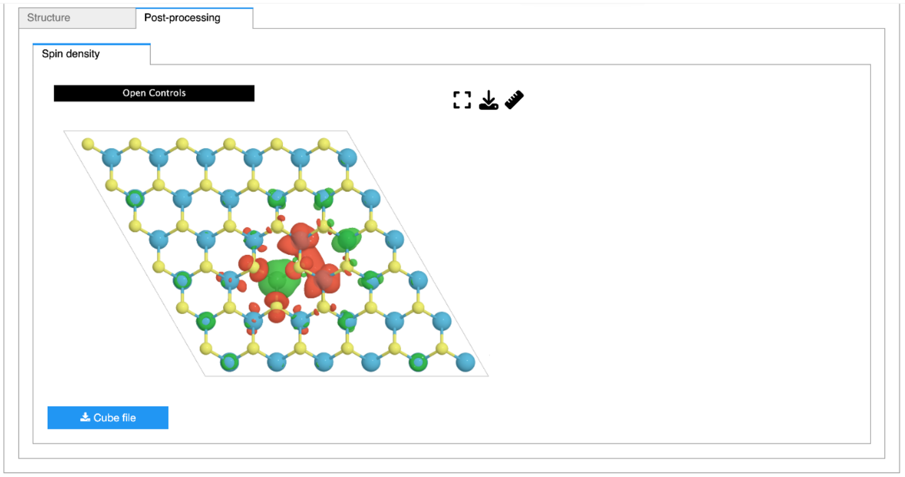

Figure S12. The result panels for the aiidalab-qe-pp plugin.

Bader charge analysis

Bader analysis partitions the total charge density into zero-flux atomic basins, providing chemically intuitive net charges that are invaluable for quantifying charge transfer, oxidation states, and catalytic activity[46]. The Bader plugin displays the per-atom charges in an interactive table alongside the three-dimensional structural view, as shown in Fig. S13.

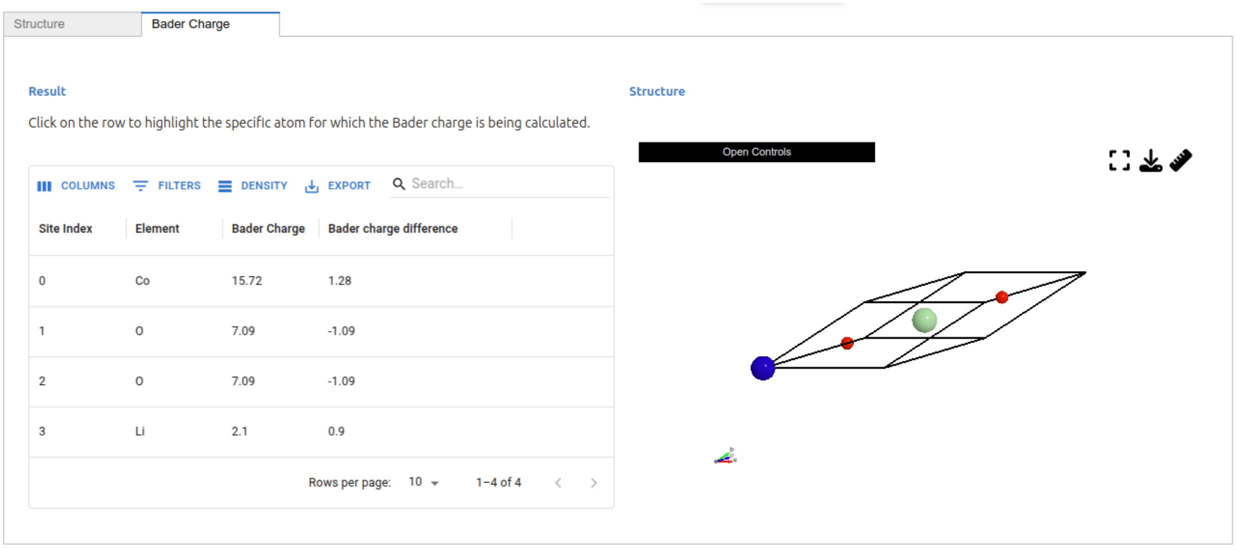

Figure S13. The result panels for the Bader charge plugin.

In-app guides

To streamline the use of the app by domain experts unfamiliar with the underlying simulation codes, we developed a system of internal guides/tutorials (Fig. S14). On selection, the activated guide injects additional information on adjacent widgets and controls, as well as a set of actions for users to take to complete the relevant steps involved in building, submitting, and visualizing results of the workflow pertaining to the subject of the guide. The guide system was developed with the plugin infrastructure in mind, allowing plugin developers to latch onto existing guide hooks, as well as introduce additional hooks within their plugin-specific components to provide description and tasks focused on the plugin's features (Fig. S14d-e). Encouraging further exploration of the app, we also provide post-guide exercises for users to try, including external resources for further learning (Fig. S14f).

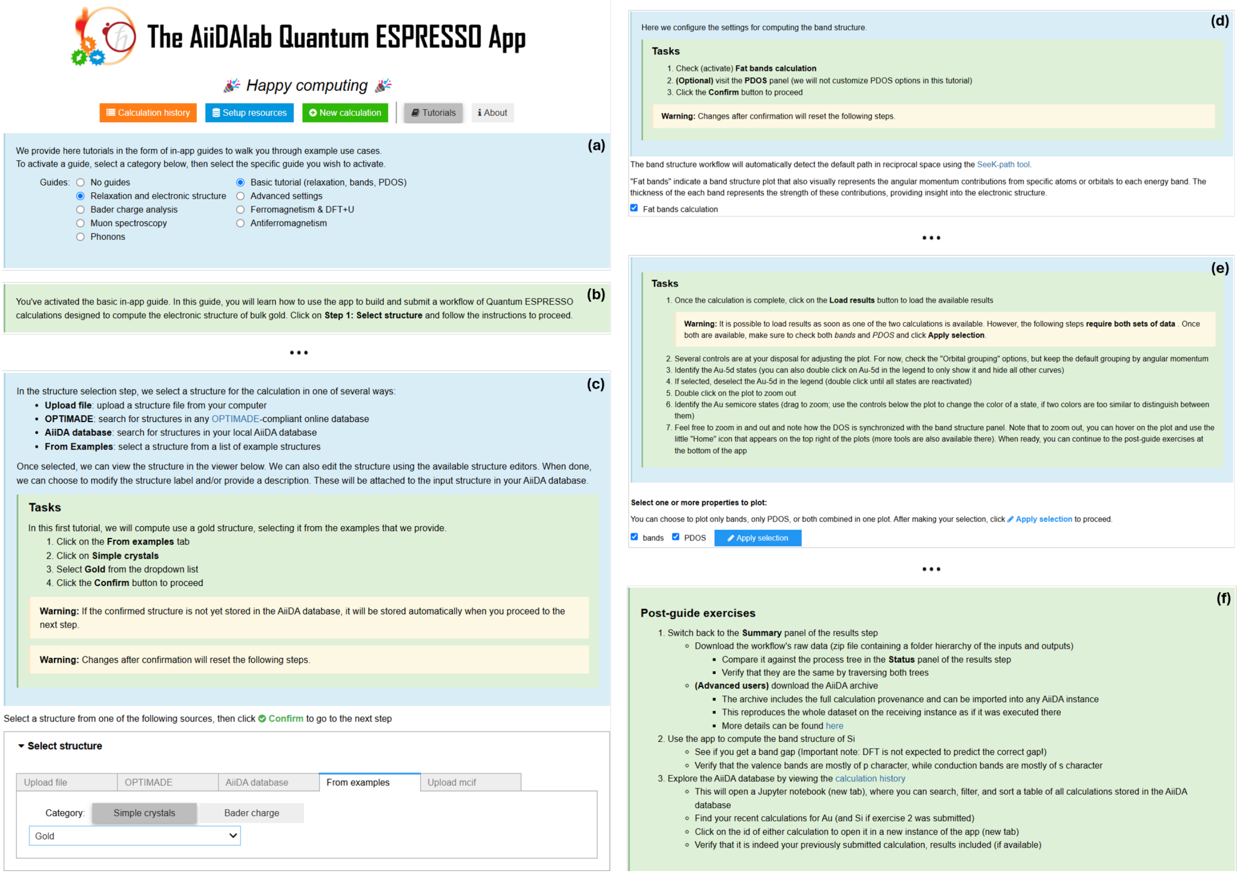

Figure S14. The QE app's in-app guides, here showing (a) the guide selection panel, (b) the guide banner, (c) a built-in structure panel guide, electronic structure plugin guides for (d) parameter settings and (e) results analysis, and (f) post-guide wrap up and further exploration.

Utilities

Calculation history

To facilitate better job management, the QE app provides a calculation history interface that allows users to efficiently search and manage their computational jobs run by the app. Fig. S15 illustrates the calculation history page. The page displays a table of all jobs in the database, along with various filtering options to help finding specific jobs. The table includes columns for the job ID, structure, creation time, state, label, relax type, and properties associated with each job. Additionally, each row in the table provides links to delete the respective job or to download its files. Users can filter the displayed data using various filters: properties, job state, label search, and date range. On the delete page, the details of a specific job are shown. Before deletion, the system checks for any dependent calculations linked to the node. If dependencies are found, the deletion is halted, and a warning is displayed. If no dependencies are present, the system asks for explicit user confirmation to ensure the action irreversibility is understood, preventing accidental deletion.

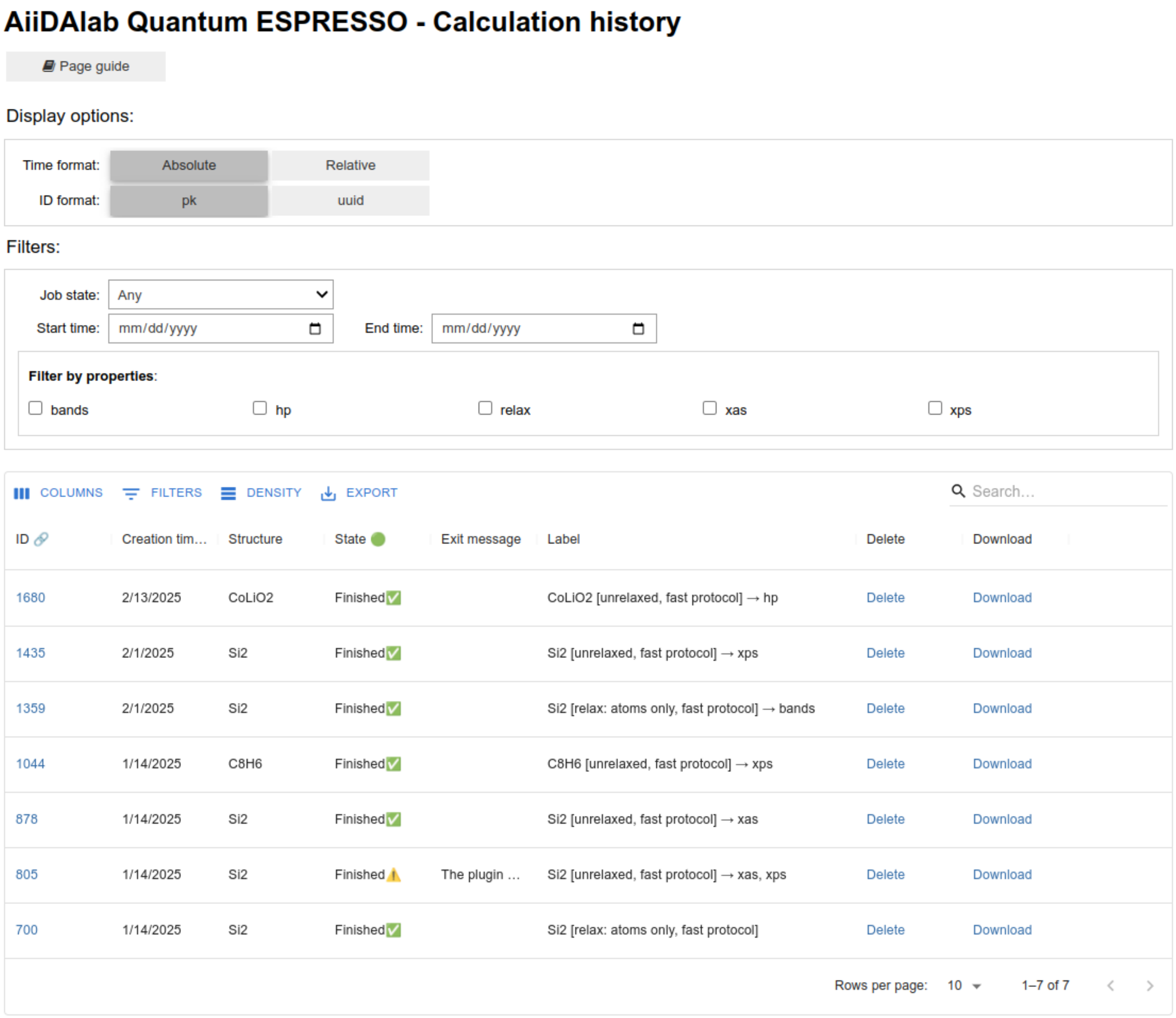

Figure S15. Calculation history interface of the QE app. The interface displays a searchable and filterable table of all previously run jobs, showing essential metadata such as job ID, structure, creation time, state, and associated properties. Users can edit job labels, delete or download results, and apply filters based on job type, date range, and computed properties. The interface aids in managing and tracking simulation workflows efficiently.

Plugin management

The QE app also includes a plugin management page that simplifies how users find and manage their plugins, making it easier to customize and enhance their computational environment. Users can browse and install plugins available in the official AiiDAlab Quantum ESPRESSO Plugin registry directly from the app. Developers are encouraged to register their plugins in this registry, which includes essential information such as the plugin's name, a link to its Git repository, documentation, and a categorization of its functions.

Figure S16 showcases the plugin management interface in the app. Once a plugin is integrated into the registry, it is automatically listed on the app's management page, making it accessible to every AiiDAlab user. This infrastructure not only aids developers in sharing their code and expertise with the community, but also allows them to maintain complete control over the development process of their GUIs.

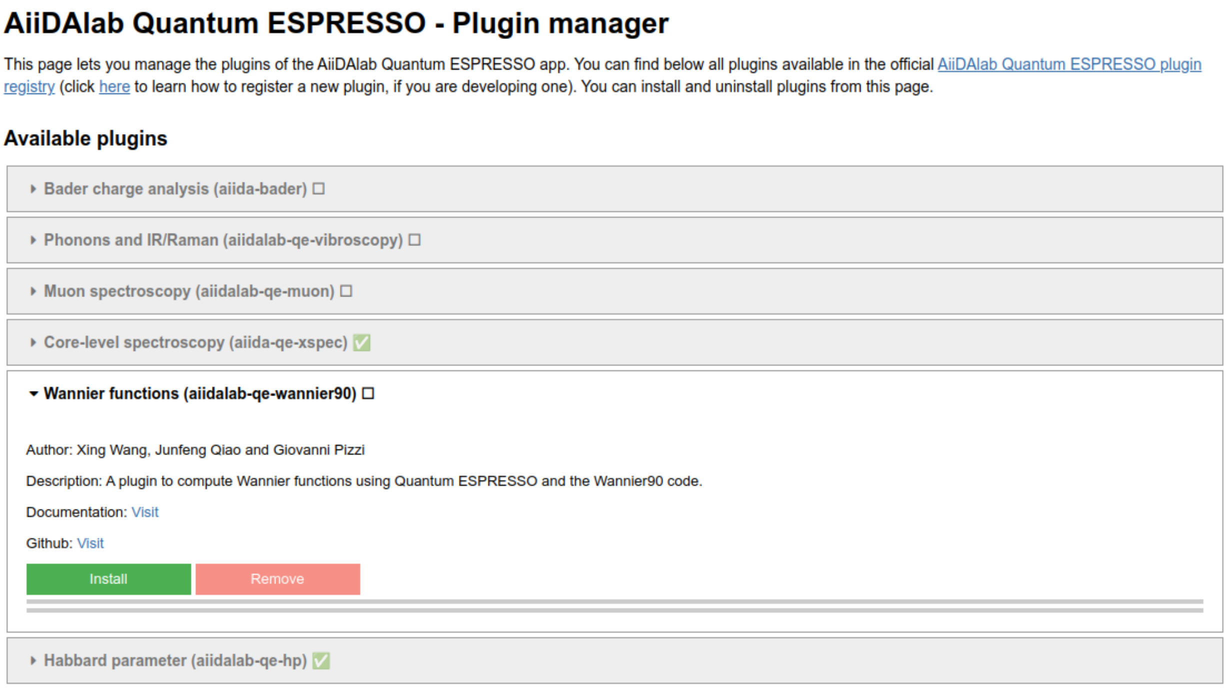

Figure S16. Plugin management interface of the QE app. The interface provides a centralized view of all available Quantum ESPRESSO plugins from the official registry, allowing users to browse, install, or remove plugins with a single click. Each plugin entry includes metadata such as name, description, author(s), links to documentation and source code, and status indicators for installed plugins. This system supports modular extension of the app, enabling users to tailor their simulation environment and developers to distribute and maintain their plugins efficiently.

Computational resources setup

Though not an integrated part of the app, we maintain an external notebook as part of the AiiDAlab ecosystem dedicated for managing computational resources (Fig. S17). The notebook allows users to create AiiDA codes, which reference executables on local (AiiDAlab container) or remote machines. For remote machines, the notebook provides a set of widgets for establishing parameters for the SSH connection, defining the machine itself, and specifying the code executable on the machine. The widgets represent a one-to-one UI to AiiDA's resource creation API. The notebook also provides a table of available codes on the container. Users can browse through the table (using filters for convenience) and optionally hide codes to exclude them from code selectors in the app (in step 3).

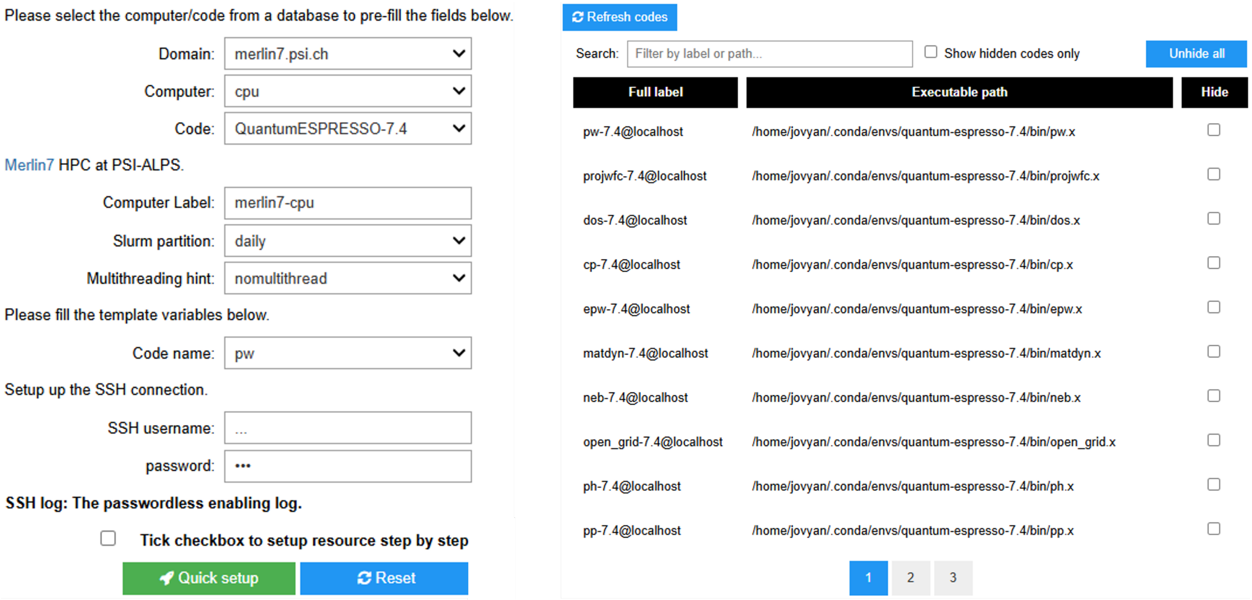

Figure S17. External resource management notebook allowing users to define AiiDA references to remote executables. On the left, users can select pre-defined remote machines and select from a set of common QE codes. Advanced users more familiar with AiiDA can always opt for full control over the process by ticking the provided checkbox. On the right, a list of available codes in the current AiiDA instance is provided to users. Codes may be marked as hidden, thus excluding them from code selectors in the app (step 3).

App examples

We provide a `Download Examples` page to help users quickly understand the capabilities of the app and its available plugins. Users can download predefined examples (such as electronic band structures, phonons, muons spectroscopies, and more), load them directly into the app, and explore the complete workflow. By inspecting each step, especially the Results Panel, users gain a clear and comprehensive understanding of what the app has to offer. This allows them to quickly assess whether the tool meets their specific needs. As shown in Fig. S18, users land on a page where they can browse a descriptive list of available examples.

Multiple examples can be selected at once. By clicking the Import button, the chosen examples are automatically downloaded and imported into the AiiDA database. Once imported, the examples appear in the Calculation History (see Fig. S15) each with a recognizable label that corresponds to the example list. From there, users can open and inspect each example in detail.

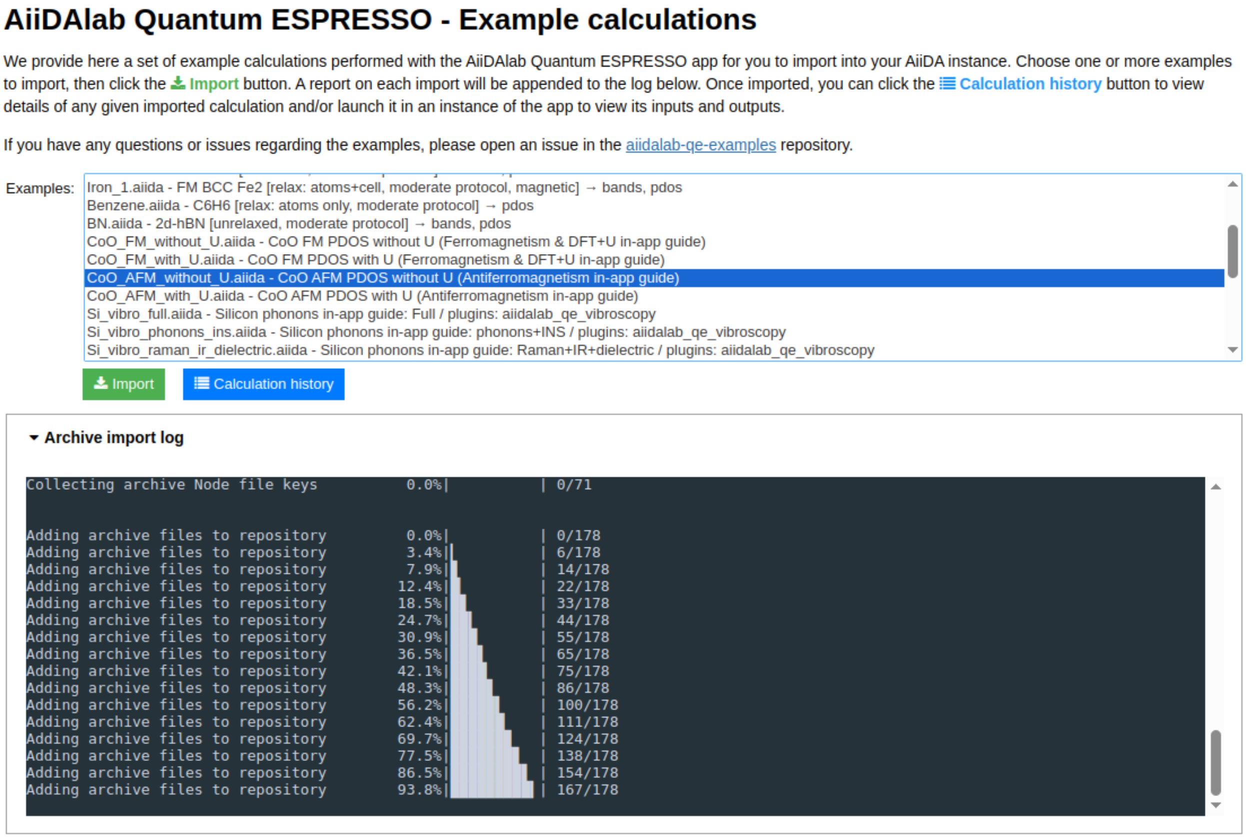

Figure S18. The *Download Examples* page allows users to select one or more predefined examples from a descriptive *Examples* list. By clicking the "Import" button, the selected examples are automatically downloaded and imported into the AiiDA database. They are then available in the Calculation History with recognizable labels, ready to be opened and explored.

Model-View-Controller

The QE app follows a standard **Model-View-Controller** (MVC) design patten, where the traits of **View-Controller** widgets - here referring to `traitlets` traits - are maintained in `Model` subclasses, where `Model` is a simple extension of the `traitlets.HasTraits` base class to provide `Mixin` support for ease of further extension. The different components of the app are connected via a network of models, leveraging both the **Observer** and **Moderator** design patterns to facilitate data transfer by using the trait observation and linking functionalities provided in the `traitlets` library.

Changes in the state of a given model propagate through the model network, triggering subscribed observables to update local states that are observed by the user interface. This design sets up clear communication between user interactions and the app's data model, from workflow setup, to submission, to monitoring, and analyzing of results.

To simplify development and maintenance, the model network is constructed with a set of dedicated trait-aware `Mixin` classes, providing both specific traits and functionality, for example the `HasInputStructure` mixin with its `input_structure` trait and `has_pbc` property method, or the `HasProcess` mixin providing a `process_uuid` and a host of dedicated methods to interact with AiiDA processes.

The separation of responsibilities provided by the MVC design pattern simplifies system and integration testing by dedicating separate testing suites for UI and models. More importantly, decoupling the UI from the data model provides clear patterns for development of the front-end and more strictly defines the app's source of truth in the model network.

Lastly, the separation of the UI and the data model enables the implementation of lazy-loading techniques and mechanisms in the UI, ensuring that only those widgets that are required to be loaded at a given moment are loaded, with others only loaded on user demand, usually by means of opening an accordion panel, or switching to an unvisited tab. Such built-in `ipywidgets` container widgets have been equipped with loading (spinner) widgets via a generic

`LoadingWidget` class to improve communication of UI loading to users. Overall, the implementation of lazy-loading techniques has significantly improved the loading time of the app by deferring/distributing minor loading times to each widget.

References

1. de Miranda Nascimento, G. *et al.* Accurate and efficient protocols for high-throughput first-principles materials simulations. *arXiv* **2504.03962** (2025).
2. de Réotier, P. D. & Yaouanc, A. Muon spin rotation and relaxation in magnetic materials. *J. Physics: Condens. Matter* **9**, 9113 (1997).
3. Möller, J. S., Ceresoli, D., Lancaster, T., Marzari, N. & Blundell, S. J. Quantum states of muons in fluorides. *Phys. Rev. B* **87**, 121108 (2013).
4. Bernardini, F., Bonfà, P., Massidda, S. & De Renzi, R. Ab initio strategy for muon site assignment in wide band gap fluorides. *Phys. Rev. B* **87**, 115148 (2013).
5. Bonfà, P., Frassineti, J., Isah, M. M., Onuorah, I. J. & Sanna, S. UNDI: An open-source library to simulate muon-nuclear interactions in solids. *Comput. Phys. Commun.* **260**, 107719 (2021).
6. Bonacci, M., Onuorah, I. J., Renzi, R. D., Bonfà, P. & Pizzi, G. aiidalab-qe-muon. https://github.com/aiidalab/aiidalab-qe-muon (2023).
7. Onuorah, I. J. *et al.* Automated computational workflows for muon spin spectroscopy. *Digit. Discov.* **4**, 523–538 (2025).
8. Onuorah, I. J. *et al.* positivemuon/aiida-muon: v1.0.3, 10.5281/zenodo.14594493 (2025).
9. Onuorah, I. J. *et al.* positivemuon/aiida-impuritysupercellconv: v1.0.1, 10.5281/zenodo.14594496 (2025).
10. Onuorah, I. J. *et al.* Automated computational workflows for muon spin spectroscopy. *Mater. Cloud Arch.* **4**, 2024.132 (2024).
11. Krishna, D. N. G. & Philip, J. Review on surface-characterization applications of x-ray photoelectron spectroscopy (xps): Recent developments and challenges. *Appl. Surf. Sci. Adv.* **12**, 100332 (2022).
12. Triguero, L., Pettersson, L. & Ågren, H. Calculations of near-edge x-ray-absorption spectra of gas-phase and chemisorbed molecules by means of density-functional and transition-potential theory. *Phys. Rev. B* **58**, 8097 (1998).
13. Cavigliasso, G. & Chong, D. P. Accurate density-functional calculation of core-electron binding energies by a total-energy difference approach. *J. Chem. Phys.* **111**, 9485–9492 (1999).
14. Walter, M., Moseler, M. & Pastewka, L. Offset-corrected δ-kohn-sham scheme for semiempirical prediction of absolute X-ray photoelectron energies in molecules and solids. *Phys. Rev. B* **94**, 041112 (2016).
15. aiida-qe-xspec: An AiiDA plugin designed for core-level spectroscopy calculations using Quantum ESPRESSO. https://github.com/aiidaplugins/aiida-qe-xspec (2025).
16. van Bokhoven, J. A. & Lamberti, C. *X-Ray Absorption and X-Ray Emission Spectroscopy*, vol. 1-2 (Wiley, 2016).
17. Klein, B. P., Hall, S. J. & Maurer, R. J. The nuts and bolts of core-hole constrained ab initio simulation for K-shell x-ray photoemission and absorption spectra. *J. Physics: Condens. Matter* **33**, 154005, 10.1088/1361-648X/abdf00 (2021).
18. Shirley, E., Pettersson, L. & Prendergast, D. *International Tables for Crystallography, Volume I, X-ray Absorption Spectroscopy and Related Techniques*, chap. Core-hole potentials and related effects, 1–9 (International Tables for Crystallography: Volume I, X-ray Absorption Spectroscopy, Internation Union of Crystallography, Chester, 2021).
19. te Velde, G. *et al.* Chemistry with ADF. *J. Comput. Chem.* **22**, 931–967 (2001).
20. Neese, F. The orca program system. *WIREs Comput. Mol. Sci.* **2**, 73–78 (2012).
21. Rehr, J. J., Kas, J. J., Vila, F. D., Prange, M. P. & Jorissen, K. Parameter-free calculations of X-ray spectra with FEFF9. *Phys. Chem. Chem. Phys.* **12**, 5503–5513 (2010).

22. Schwarz, K., Blaha, P. & Madsen, G. Electronic structure calculations of solids using the wien2k package for material sciences. *Comput. Phys. Commun.* **147**, 71–76 (2002). Proceedings of the Europhysics Conference on Computational Physics Computational Modeling and Simulation of Complex Systems.

23. Giannozzi, P. *et al.* Quantum ESPRESSO toward the exascale. *J. Chem. Phys.* **152**, 154105, 10.1063/5.0005082 (2020).

24. Gougoussis, C., Calandra, M., Seitsonen, A. P. & Mauri, F. First-principles calculations of x-ray absorption in a scheme based on ultrasoft pseudopotentials: From α-quartz to high-T_c compounds. *Phys. Rev. B* **80**, 075102 (2009).

25. Bunău, O. & Calandra, M. Projector augmented wave calculation of x-ray absorption spectra at the $L_{2,3}$ edges. *Phys. Rev. B* **87**, 205105 (2013).

26. Prendergast, D. & Galli, G. X-ray absorption spectra of water from first principles calculations. *Phys. Rev. Lett.* **96**, 215502, 10.1103/PhysRevLett.96.215502 (2006).

27. Marzari, N. & Vanderbilt, D. Maximally localized generalized Wannier functions for composite energy bands. *Phys. Rev. B* **56**, 12847 (1997).

28. Marzari, N., Mostofi, A. A., Yates, J. R., Souza, I. & Vanderbilt, D. Maximally localized Wannier functions: Theory and applications. *Rev. Mod. Phys.* **84**, 1419–1475, 10.1103/RevModPhys.84.1419 (2012).

29. Marrazzo, A. *et al.* Wannier-function software ecosystem for materials simulations. *Rev. Mod. Phys.* **96**, 045008 (2024).

30. Pizzi, G. *et al.* Wannier90 as a community code: new features and applications. *J. Physics: Condens. Matter* **32**, 165902 (2020).

31. Qiao, J., Pizzi, G. & Marzari, N. Projectability disentanglement for accurate and automated electronic-structure Hamiltonians. *npj Comput. Mater.* **9**, 208 (2023).

32. aiida-wannier90-workflows. https://github.com/aiidateam/aiida-wannier90-workflows (2025).

33. Damle, A., Lin, L. & Ying, L. Compressed representation of Kohn–Sham orbitals via selected columns of the density matrix. *J. Chem. Theory Comput.* **11**, 1463–1469 (2015).

34. Rourke, P. & Julian, S. Numerical extraction of de Haas–van Alphen frequencies from calculated band energies. *Comput. Phys. Commun.* **183**, 324–332 (2012).

35. Wang, X. Weas widget. https://github.com/superstar54/weas-widget (2025).

36. Anisimov, V., Zaanen, J. & Andersen, O. Band theory and Mott insulators: Hubbard U instead of Stoner I. *Phys. Rev. B* **44**, 943 (1991).

37. Liechtenstein, A., Anisimov, V. & Zaanen, J. Density-functional theory and strong interactions: Orbital ordering in Mott-Hubbard insulators. *Phys. Rev. B* **52**, R5467 (1995).

38. Dudarev, S., Botton, G., Savrasov, S., Humphreys, C. & Sutton, A. Electron-energy-loss spectra and the structural stability of nickel oxide: An LSDA+U study. *Phys. Rev. B* **57**, 1505 (1998).

39. Campo, V. L. & Cococcioni, M. Extended DFT+ U+ V method with on-site and inter-site electronic interactions. *J. Physics: Condens. Matter* **22**, 055602 (2010).

40. Cococcioni, M. & de Gironcoli, S. Linear response approach to the calculation of the effective interaction parameters in the LDA+U method. *Phys. Rev. B* **71**, 035105 (2005).

41. Timrov, I., Marzari, N. & Cococcioni, M. Hubbard parameters from density-functional perturbation theory. *Phys. Rev. B* **98**, 085127 (2018).

42. Timrov, I., Marzari, N. & Cococcioni, M. Self-consistent Hubbard parameters from density-functional perturbation theory in the ultrasoft and projector-augmented wave formulations. *Phys. Rev. B* **103**, 045141 (2021).

43. Bastonero, L. *et al.* First-principles Hubbard parameters with automated and reproducible workflows. *npj Comput. Mater.* **11**, 183, 10.1038/s41524-025-01685-4 (2025).

44. AiiDAteam. AiiDA plugin for the first-principles calculation of Hubbard parameters. https://github.com/aiidateam/aiida-hubbard (2025).

45. Timrov, I., Marzari, N. & Cococcioni, M. HP–A code for the calculation of Hubbard parameters using density-functional perturbation theory. *Comput. Phys. Commun.* **279**, 108455 (2022).

46. Tang, W., Sanville, E. & Henkelman, G. A grid-based Bader analysis algorithm without lattice bias. *J. Physics: Condens. Matter* **21**, 084204 (2009).
